# The Capability to Undergo ACSL4-Mediated Ferroptosis Is Acquired During Brown-like Adipogenesis and Affected by Hypoxia

**DOI:** 10.3390/cells14161247

**Published:** 2025-08-13

**Authors:** Markus Mandl, Elisabeth Heuboeck, Peter Benedikt, Florian Huber, Olga Mamunchak, Sonja Grossmann, Michaela Kotnik, Esma Hamzic-Jahic, Charnkamal Singh Bhogal, Anna-Maria Lipp, Edeltraud Raml, Werner Zwerschke, Martin Wabitsch, Jakob Voelkl, Andreas Zierer, David Bernhard

**Affiliations:** 1 Institute for Physiology and Pathophysiology, Department of Pathophysiology, Johannes Kepler University Linz, Krankenhausstrasse 5, 4020 Linz and Altenberger Strasse 69, 4040 Linz, Austriaesma.hamzic-jahic@jku.at (E.H.-J.); charnkamal.bhogal@jku.at (C.S.B.); david.bernhard@jku.at (D.B.); 2 Clinical Research Institute for Cardiovascular and Metabolic Diseases, Medical Faculty, Johannes Kepler University Linz, Altenberger Strasse 69, 4040 Linz, Austria; peter.benedikt@kepleruniklinikum.at (P.B.); florian.huber@kepleruniklinikum.at (F.H.); olga.mamunchak2@kepleruniklinikum.at (O.M.); andreas.zierer@kepleruniklinikum.at (A.Z.); 3 Department of Cardiac, Vascular and Thoracic Surgery, Kepler University Hospital Linz, 4020 Linz, Austria; 4 Division of Cell Metabolism and Differentiation Research, Research Institute for Biomedical Aging Research, University of Innsbruck, 6020 Innsbruck, Austria; sonja.grossmann@uibk.ac.at (S.G.); werner.zwerschke@uibk.ac.at (W.Z.); 5 Core Facility for Cytometry, Center for Medical Research, Johannes Kepler University Linz, 4040 Linz, Austria; anna-maria.lipp@jku.at (A.-M.L.); edeltraud.raml@jku.at (E.R.); 6 Department of Pediatrics and Adolescent Medicine, Ulm University Medical Center, 89075 Ulm, Germany; martin.wabitsch@uniklinik-ulm.de; 7 German Center for Child and Adolescent Health (DZKJ), Partner Site Ulm, 89075 Ulm, Germany; 8 Institute for Physiology and Pathophysiology, Department of Physiology, Johannes Kepler University Linz, 4020 Linz, Austria; jakob.voelkl@jku.at; 9 Department of Nephrology and Medical Intensive Care, Charité-Universitaetsmedizin Berlin, Corporate Member of Freie Universitaet Berlin and Humboldt Universitaet zu Berlin, 13353 Berlin, Germany; 10 German Centre for Cardiovascular Research (DZHK), Partner Site Berlin, 13347 Berlin, Germany

**Keywords:** hypoxia, ferroptosis, epicardial adipose tissue

## Abstract

Adipose tissue enlargement in obesity leads to hypoxia, which may promote premature aging. This study aimed to understand the hypoxic response in 3D cultures of SGBS cells, a model for brown-like adipose tissue expressing uncoupling protein 1 (UCP1). Single-nucleus RNA sequencing of SGBS organoids revealed a heterogeneous composition and sub-population-specific responses to hypoxia. The analysis identified a cluster of transcriptional repression, indicating dying cells, and implied a role of ferroptosis in this model. Further experiments with SGBS cells and white adipose tissue-derived stem/progenitor cells showed that Acyl-CoA synthetase long-chain family member 4 (ACSL4), a key enzyme in ferroptosis, is expressed only in the presence of browning factors. Hypoxia downregulated ACSL4 protein in SGBS organoids but induced an inflammaging phenotype. Analysis of brown-like epicardial adipose tissue from cardiac surgery patients revealed a significant positive correlation of ACSL4 mRNA with UCP1 and hypoxia-inducible pro-inflammatory markers, while ACSL4 protein appeared to be inversely correlated. In conclusion, this study demonstrates that adipocytes’ capability to undergo ACSL4-mediated ferroptosis is linked to brown-like adipogenesis, suggesting an opportunity to modulate ferroptotic signaling in adipose tissue. The dual role of hypoxia by inhibiting ACSL4 but promoting inflammaging indicates a relationship between ferroptosis and aging that warrants further investigation.

## 1. Introduction

Obesity is regarded as a state of accelerated aging [1,2] and affects one in eight adults worldwide [3]. Adipose tissue (AT) enlargement in obesity is either achieved by the increase in adipocyte number (hyperplasia) and/or -size (hypertrophy) [4,5]. Hyperplasia includes the proliferation and differentiation of adipogenic stem/progenitor cells (ASCs) into mature adipocytes, a process known as adipogenesis [5,6]. Adipogenesis is initiated by signaling molecules such as hormones leading to the expression of key transcription factors like the CCAAT/enhancer-binding protein beta (C/EBPβ) and peroxisome proliferator-activated receptor gamma (PPARγ), among others [7,8]. In contrast, hypertrophy is strongly associated with adipose tissue dysfunction due to the onset of hypoxia, mitochondrial impairment, oxidative stress (i.e., reactive oxygen species (ROS)) and increased cell death [4,5,9]. Emerging evidence demonstrates that targeting AT aging holds promise to mitigate age-related physical decline and to extend health span [10,11].

Adipose tissue depots can be distinguished depending on the anatomical location and function. White AT (WAT) serves primarily as triglyceride storage whereas brown AT (BAT) generates heat by thermogenesis due to the expression of uncoupling protein 1 (UCP1), a protein that disengages the mitochondrial proton gradient from adenosine triphosphate (ATP) production [11,12]. Studies have shown that the development of BAT is mediated by PPARγ agonists (e.g., Rosiglitazone) throughout adipogenesis [13,14]. During aging, WAT is redistributed leading to increased visceral mass, while subcutaneous AT declines [11]. Epicardial AT (EAT) is a unique visceral fat depot adjacent to the heart, which undergoes brown-to-white trans-differentiation over time, thereby losing its thermogenic capability [15]. These observations imply depot-specific effects of aging [16].

Hypoxia (defined as limited oxygen availability) is considered a driver of aging in various models [17]. On the molecular level, hypoxia is sensed by the hypoxia-inducible factor (HIF) pathway. Prolylhydroxylase-domain enzymes (PHDs) become inactive due to the lack of O_2_ or by ROS-mediated Fe^2+^ oxidation, thereby preventing HIF-α subunits (i.e., HIF-1α, HIF-2α, HIF-3α) from degradation by the ubiquitin/proteasome pathway [18,19,20]. Heterodimerization of HIF-α and HIF-β subunits (i.e., HIF-1β, HIF-2β) initiates broad tissue-specific transcriptional changes including metabolic adaptations [19]. Hypoxia is known to promote cellular senescence, a hallmark of aging [17]. Senescent cells are non-proliferative, show a high lysosomal activity (i.e., Senescence-associated β-Galactosidase) and secrete various pro-inflammatory mediators, which are collectively referred to as the senescence-associated secretory phenotype (SASP) [2,5,11,21]. Studies have shown that mature adipocytes might acquire a senescence phenotype after differentiation including SASP [22,23,24]. However, the role of hypoxia in this context is incompletely understood.

The objective of this study was to explore the effects of hypoxia in a human organoid model of adipose tissue by single-nucleus RNA sequencing (snRNA seq) in an unbiased manner. Our study reveals that the expression of Acyl-CoA synthetase long-chain family member 4 (ACSL4), an indispensable enzyme for ferroptosis execution [25], is linked to brown-like adipogenesis. Ferroptosis is a specific form of programmed cell death that is mediated by ROS- and iron-dependent phospholipid peroxidation [26]. ACSL4 is a driver of ferroptosis and catalyzes the reaction of Coenzyme-A (CoA) with polyunsaturated fatty acids (PUFAs), such as arachidonic acid, thereby generating Arachidonyl-CoA (AA-CoA), among others [25]. Subsequently, AA-CoA is incorporated into phospholipids, which are finally converted into lipid peroxides that accumulate in cellular membranes, leading to ferroptotic membrane ruptures [25]. On the other site, antioxidant systems (e.g., the Xc−/GSH/GPX4 axis) protect the cell from undergoing ferroptosis by counteracting lipid peroxidation [27]. Therefore, an imbalanced redox homeostasis promotes ferroptosis [26] (for a comprehensive overview of ferroptosis, the reader is referred to Refs. [26,28]). In adipocytes, ferroptosis is largely unexplored. Recent reports suggest a link between ferroptosis, obesity and aging as these conditions are highly related to oxidative stress [29,30,31].

In our study, ACSL4 was only expressed in the presence of browning factors (i.e., Triiodothyronine (T3), Cortisol, and Rosiglitazone) during adipogenesis. In addition, hypoxia-mediated ACSL4 inhibition was associated with SASP induction. These findings imply the regulation of ferroptosis by molecules affecting browning and suggest a connection between ferroptosis and inflammaging in adipose tissue.

## 2. Materials and Methods

### 2.1. Ethics Statement and Origin of Biological Materials

The human Simpson–Golabi–Behmel Syndrome (SGBS) cells were kindly provided by Prof. Dr. Martin Wabitsch (Department of Pediatrics and Adolescent Medicine, Division of Pediatric Endocrinology and Diabetes, Ulm University Medical Center, Ulm, Germany) [32,33]. Human Adipogenic stem/progenitor cells (ASCs) isolated from white subcutaneous adipose tissue samples of one male and one female donor were kindly provided by Prof. Dr. Werner Zwerschke (Division of Cell Metabolism and Differentiation Research, Research Institute for Biomedical Aging Research, University of Innsbruck, Innsbruck, Austria) [34].

Human epicardial adipose tissue (EAT) samples were obtained from patients undergoing cardiac surgery via a full sternotomy at the University Hospital for Cardiac, Vascular and Thoracic Surgery, Linz, Austria (Head: Prof. Dr. Andreas Zierer). All patients gave their informed written consent according to the Declaration of Helsinki and as approved by the Ethics Committee of the Johannes Kepler University (JKU) Medical Faculty, Linz, Austria (EK Nr: 1231/2024). Patient characteristics are provided in Table A1.

### 2.2. Cell Culture

Human SGBS cells were cultured in Growth Medium containing DMEM/F12, HEPES (Gibco, # 31330038), 3.3 mM Biotin, 1.7 mM Panthotenat, 1% Penicillin/Streptomycin and 10% FBS (not heat-inactivated). Human WAT-derived ASCs were cultured as described in Hatzmann et al. 2021 [34]. The medium was changed 2–3 times per week and cell cultures were split at ~80% confluency.

### 2.3. Specific Activators and Inhibitors

The ferroptosis-inducer Erastin [35], the ferroptosis inhibitor Deferoxamine (DFO) [36] and the HIF-1α inhibitor TAT-Cyclo-CLLFVY (TAT-Cyclo) [37] were purchased from MedChemExpress and dissolved in DMSO. Equivalent concentrations of DMSO were used as vehicle controls throughout experiments.

### 2.4. Hypoxia Treatment

For hypoxic exposure, cells and organoids were cultured for 24 h in a 3% O_2_, balanced N_2_ and humidified atmosphere by using either a conventional cell culture incubator or the Biospherix x3 Xvivo System (Biospherix, Parish, NY, USA). The Image-iT™ Green Hypoxia Reagent (ThermoScientific, Waltham, MA, USA) was used to confirm the induction of hypoxia according to the supplier’s protocol, among others.

### 2.5. Adipogenic Differentiation of 2D Cultures

SGBS cells and WAT-derived ASCs were seeded in 6-well plates and cultured until confluent. Stock solutions for differentiation were prepared as follows in accordance with established protocols and with minor modifications [32,33,38]: Biotin—33 mM in DMEM/F12; Pantothenate—17 mM in DMEM/F12; Transferrin—1 mg/mL in DMEM/F12; Insulin—100 µM in 10 mM HCl + 1% BSA; Cortisol—13 mM in absolute ethanol; Dexamethasone—25 µM in absolute ethanol; IBMX: 200 mM in DMSO—Rosiglitazone: 28 mM in DMSO; T3—3.6 mM in dH_2_O (+NaOH). All stock solutions were sterilized by filtration, aliquoted, and stored at −80 °C. The basal medium required for brown-like differentiation (hereafter referred to as 3FC medium) contained 3.3 µM Biotin, 1.7 µM Pantothenate, 1% Penicillin/Streptomycin, 0.01 mg/mL Transferrin, 20 nM Insulin, 100 nM Cortisol and 0.2 nM T3 in DMEM/F12. To induce brown-like adipogenic differentiation, 3FC medium was supplemented with 25 nM Dexamethasone, 250 µM IBMX and 2 µM Rosiglitazone (referred to as Quick Diff medium hereafter). For white differentiation Cortisol, T3 and Rosiglitazone were omitted in the basal medium and differentiation mixture, respectively. The adipogenic cocktail was applied for 4 days. Subsequently, cells were differentiated until day 12 in corresponding basal medium. The medium was exchanged every 2–3 days.

### 2.6. SGBS Organoid Model

SGBS cells cultured in 75 cm^2^ flasks were harvested by trypsinization (TrypLE, Gibco, Waltham, MA, USA) and counted using the Countess II system (ThermoFisher Scientific). Next, SGBS cells were seeded at a density of 2 × 10^4^ cells per well in 96-well round-bottom low attachment plates (Nunclon™ Sphera™ 96-Well, ThermoFisher Scientific) in a total volume of 100 µL Growth Medium. To avoid evaporation of cell culture medium, wells on the edges were filled with 200 µL PBS per well. Cells were incubated overnight (37 °C, 5% CO_2_, humidified atmosphere) to enable aggregation. Next, 50 µL of supernatant per well was carefully removed with a multichannel pipette and spheroids were washed with 200 µL 3FC medium per well. Adipogenic differentiation was initiated by addition of 200 µL Quick Diff medium per well followed by incubation for 4 days. Depending on the type of experiment, undifferentiated (i.e., non-induced) spheroids were maintained in 3FC medium to serve as the control. It is important to note that no medium exchange was performed during this initiation period. On day 4, 150 µL of supernatant per well was carefully removed and cells were washed with 150 µL 3FC medium per well. Subsequently, cells were treated with 150 µL/well 3FC medium and incubated up to day 12 of differentiation. The 3FC medium was replaced every 2nd or 3rd day (200 µL/well). Differentiating organoids were monitored on a regular basis by microscopy. On day 12, organoids were harvested and analyzed or treated depending on the purpose of the experiment.

### 2.7. Measurement of Organoid Size

Brightfield images were taken using the Cytation 7 System (Agilent BioTek) and imported into the *Fiji* software (Version 1.54f; https://github.com/fiji/fiji (accessed on 9 August 2025)). To measure organoid size (i.e., diameter and area), the jython macro *OrgM* was applied (Version 1.0; https://github.com/neuroeddu/OrgM (accessed on 9 August 2025)). Organoid volume was calculated assuming a spherical shape and normalized to undifferentiated controls.

### 2.8. Assessment of Intracellular Lipid Accumulation

Intracellular lipids were detected by Oil Red O staining whereas the staining procedure was slightly modified dependent on the biological sample. Organoids were washed with PBS and fixed with 4% PFA/PBS for 15 min at room temperature. Subsequently, organoids were washed with 70% isopropyl alcohol/PBS and stained with 100 µL/well Oil Red O solution (Sigma Aldrich, St. Louis, MO, USA). After 30 min, the dye was removed and organoids were washed twice with PBS. Finally, images were taken with the Cytation 7 System (8× magnification, upright position; Agilent BioTek, Santa Clara, CA, USA). Two-dimensional (2D) cell cultures of human ASCs were fixed with 4% PFA/PBS for 1 h at room temperature, washed with 60% isopropyl alcohol and stained with Oil Red O for 1 h at room temperature as described [8]. Finally, cells were washed with PBS and images were taken using an inverse cell culture microscope.

### 2.9. Measurement of Cell Viability (i.e., Redox Capacity) in Organoids

Resazurin (MedChemExpress, Monmouth Junction, NJ, USA) was dissolved in PBS (1% *w*/*v*) and sterilized by filtration (0.2 µm filter). Next, 5 µL of Resazurin solution was added to each organoid in a total volume of 200 µL. An equal volume of cell culture medium containing 5 µL Resazurin solution served as a blank. Organoids were incubated overnight (37 °C, 5% CO_2_, humidified atmosphere) to convert Resazurin into Resorufin [39]. Subsequently, 150 µL of cell culture supernatant per well was transferred into an ELISA plate and measured at 595 nm and 570 nm. Resazurin turnover was expressed as ratio OD_570_/OD_595_.

### 2.10. Assessment of Senescence-Associated Beta-Galactosidase (SA β-Gal) Activity

SA β-Gal activity was determined in cryo-sections (8 µm) of PFA-fixed organoids and whole organoids. The staining procedure was carried out as described in Ref. [40] with minor modifications. Cryo-sections were imaged using the Olympus VS200 ASW Research Slide Scanner (Olympus, Tokyo, Japan). Subsequently, microphotographs were analyzed as outlined in Ref. [41] and ImageJ software (Version 1.54; National Institutes of Health, Bethesda, MD, USA). Imaging of whole organoids was performed with the Cytation 7 System (Agilent BioTek).

### 2.11. Nuclei Isolation from SGBS Organoids

Isolation of nuclei was performed as described in the demonstrated protocol “Nuclei Isolation from Cell Suspensions & Tissues for Single Cell RNA Sequencing” by 10× Genomics with modifications (https://cdn.10xgenomics.com/image/upload/v1660261285/support-documents/CG000124_Demonstrated_Protocol_Nuclei_isolation_RevF.pdf (accessed on 9 August 2025)). In detail, SGBS organoids were washed with cold PBS (200 µL/well) and 100 µL of Lysis Buffer (10 mM Tris-HCl pH 7.4, 10 mM NaCl, 3 mM MgCl_2_, NP40 Substitute 0.1%; sterilized by filtration) was added per well, followed by 5 min incubation at room temperature. Organoids were collected (usually ~6–10 organoids per sample), transferred into a 1.5 mL tube and centrifuged (10 min, 600 rcf, 4 °C). The supernatant was removed (except ~50 µL) and 50 µL of TrypLE was added. Organoids were digested on ice and disintegrated using a sterile pistil until the suspension became cloudy. Next, 1 mL of Nuclei Wash & Resuspension Buffer (1% BSA/PBS supplemented with 0.2 U/µL RiboLock RNase Inhibitor (ThermoScientific); sterilized by filtration) was added. The solution was cleared by filtration (FlowMi^®^ cell strainer, 40 µm pore size; BelArt products, Wayne, NJ, USA) and centrifuged (10 min, 600 rcf, 4 °C). The supernatant was removed carefully and nuclei were washed with 1 ml Nuclei Wash & Resuspension Buffer. Nuclei were resuspended in 30 µL Nuclei Wash & Resuspension Buffer and counted with the Countess II System (ThermoFisher Scientific). To stain DNA, 5 µL of nuclei suspension was mixed with 5 µL Hoechst staining solution (dilution 1:2500 in PBS) prior to counting. For single-nucleus RNA sequencing, the concentration was adjusted to ~1 × 10^6^ nuclei/mL.

### 2.12. Flow Cytometry of Isolated Nuclei

Isolated nuclei were resuspended in 0.1 µM sterile-filtered staining buffer containing 1× PBS, at pH 7.4 (VWR Chemicals, Radnor, PA, USA), 0.5% BSA and 2 mM EDTA (both Sigma-Aldrich, St. Louis, MO, USA) and were stained overnight at 4 °C with Alexa Fluor 680 (AF680)-conjugated Lamin A/C antibody (Santa Cruz, sc-7292, Dallas, TX, USA). Following incubation, nuclei were washed three times at 2250× *g* for 5 min at 4 °C using the same staining buffer. DAPI (Applichem GmbH, Darmstadt, Germany) was then added to the suspension at a final concentration of 1 µg/mL. Flow cytometry analysis was performed using a Cytek^®^ Northern Lights™ instrument (Cytek Biosciences, CA, USA). Nuclei were gated using a log side scatter (SSC-A) vs. log forward scatter (FSC-A) bivariate plot, with singlets identified based on log FSC-H vs. log FSC-A. The percentage of positive events was then determined from a log DAPI-A vs. log AF680-A bivariate plot.

### 2.13. Processing of Epicardial Adipose Tissue (EAT) Samples

EAT samples were collected in 1 mL TRIzol and homogenized using the gentleMACS™ Octo Dissociator with Heaters (Miltenyi Biotec, Bergisch Gladbach, Germany) in combination with MACS C-tubes (Miltenyi Biotec, Bergisch Gladbach, Germany) (program: RNA_02). Simoultanous RNA and protein isolation was performed with the Direct-zol™ RNA Microprep Kit (Zymo Research, Irvine, CA, USA) according to the manufacturer’s protocol.

### 2.14. Gene Expression Analysis by Reverse Transcription–Quantitative PCR (RT-qPCR)

RNA isolation and cDNA synthesis were performed dependent on the biological sample and the purpose of the experiment (see also “Single-nucleus RNA sequencing (snRNA seq)”). For isolated nuclei and EAT tissue samples, the SuperScript IV Single Cell/Low Input cDNA Pre Amp Kit (ThermoScientific) was used according to the manufacturer’s guidelines with the following modifications. Herein, the RT reaction was carried out at 50 °C for 45 min followed by a 16-cycle pre-amplification phase. cDNA was diluted 1:5 or 1:10, respectively, prior to qPCR analysis. Monolayer cell cultures were washed with cold PBS and harvested using Solubilization Buffer (2 mM EDTA, 1% SDS, 40 mM Tris HCl (pH 7.5), Halt™ protease inhibitor 1:50) for simultaneous RNA/protein isolation [42]. Next, samples were mixed with TRIzol™ and RNA was extracted with the Direct-zol RNA Microprep kit (Zymo Research) as outlined in the manufacturer’s instructions. cDNA synthesis was performed using the PrimeScript™ FAST RT reagent with gDNA Eraser (Takara Bio, San Jose, CA, USA) according to the supplier’s protocol. cDNA was diluted 1:3 to 1:5 prior qPCR analysis.

Finally, gene expression was measured with the CFX96 Touch Real-Time PCR Detection System (BioRad, Hercules, CA, USA) using default settings and SYBR green chemistry (SsoAdvanced Universal SYBR^®^ Green Supermixes, BioRad). Primer sequences are provided in Supplementary Table S1. Relative quantitation of gene expression data was calculated by the (Δ)ΔC_t_ method.

### 2.15. Single-Nucleus RNA Sequencing (snRNA Seq)

snRNA-seq was performed at the Core Facility for Next Generation Sequencing at the Center for Medical Research, JKU Linz, Austria. Therefore, the Chromium Next GEM Single Cell 3’ Kit v3.1, 4rxns (10× Genomics) was used. Each sample was loaded onto the Chromium Next GEM Chip G (10× Genomics) with a targeted recovery cell number of n = 5000 cells. GEM generation and barcoding, reverse transcription, cDNA generation and library construction were performed according to the manufacturer’s protocol. cDNA quality was controlled with an Agilent Bioanalyzer 2100 (Agilent Technologies, Santa Clara, CA, USA) prior library construction. Finally, dual-indexed, single-cell libraries were pooled and sequenced in paired-end reads on a NextSeq2000 system (Illumina, San Diego, CA, USA) with a NEXTSEQ™ 1000/2000 P2 100 Cycles flow cell (Illumina) following the manufacturer’s instructions.

### 2.16. snRNA Seq Data Analysis

Raw data were inspected with CellRanger (10× Genomics) and a forced threshold of 2000 cells per sample was applied. Subsequently, the processed data were provided by the Core Facility for Next Generation Sequencing (Center for Medical Research, JKU Medical Faculty) for further analysis. In depth data analysis was performed using the SeqGeq software (version 1.7.0; Becton Dickinson & Company (BD), Franklin Lakes, NJ, USA). The data sets of both samples (i.e., normoxia and hypoxia) were concatenated and normalized (Counts per 10.000). No filtering was applied (i.e., no exclusion of events). Next, principal component (PC) analysis was carried out. The first 6 PCs were employed to perform dimensionality reduction using t-Distributed Stochastic Neighbor Embedding (t-SNE). Gating of cell populations was performed with the SeqGeq Auto-Gate function. Clusters were manually annotated based on well-described signature genes [43,44]. Differential expressed genes (DEGs) were computed as described in the software’s manual. For additional visualization, customized heatmaps were generated using the *heatmap.2*() function of the *gplots* package in R-Studio (version 2024.04.2). Venn diagrams were generated using an online tool (https://bioinformatics.psb.ugent.be/webtools/Venn/ (accessed on 9 August 2025)). Enrichment- and pathway analysis was performed using the R package *clusterProfiler* (Version 4.0), the Gene Ontology Resource (https://geneontology.org/ (accessed on 9 August 2025)) and the Reactome database (https://reactome.org/; (accessed on 9 August 2025)), respectively. Pseudotime analysis was computed with the Monocle plugin for SeqGeq.

### 2.17. Protein Determination

To gain samples for Western blotting, cells were washed with cold PBS and harvested in Solubilization Buffer (2 mM EDTA, 1% SDS, 40 mM Tris HCl (pH 7.5) and Halt™ protease inhibitor 1:50) followed by sonication. For protein quantitation, an aliquot of the sample (5–10 µL) was purified using the Compat-Able™ Assay Preparation Kit (ThermoScientific) and analyzed with the BCA Protein Assay Kit (ThermoScientific) as described in the manufacturer’s protocol.

### 2.18. Western Blotting

Protein samples (5–10 µg) were separated by polyacrylamide gel electrophoresis and blotted onto a Polyvinyl Difluoride (PVDF) membrane with the semi-dry TransBlot Turbo Transfer System (BioRad). Membranes were blocked with 5% dry milk/TBST and probed with primary antibodies (anti-ACSL4, proteintech, #22401-1-AP, 1:2500; anti-KEAP1, proteintech, #80744-1-RR, 1:2500). Subsequently, appropriate HRP-conjugated secondary antibodies were applied for 1 h at room temperature. β-Actin was determined as a loading control. Signal development was achieved using the ECL substrate (Promega, Madison, WI, USA). Finally, chemiluminescent signals were recorded with the ChemiDoc System (BioRad) and quantified using ImageLab software (Version 6.0; BioRad).

### 2.19. Measurement of Protein Oxidation

Oxidative stress was determined using the OxyBlot Protein Oxidation Detection Kit (Merck Millipore, Darmstadt, Germany) in accordance with the supplier’s guidelines.

### 2.20. Analysis of Secreted Cytokines

Semi quantitative assessment of cytokines in cell culture supernatants was performed with the RayBio^®^ C-Series Human Cytokine Antibody Array C1000 (RayBiotech, Peachtree Corners, GA, USA) as described in the manufacturer’s protocol.

### 2.21. Statistical Analysis

Statistics analysis was performed with GraphPad Prism 10 software (GraphPad Software Inc., La Jolla, CA, USA). Each experiment was performed with a minimum of n = 3 biological replicates as indicated in the corresponding figure legend. Values are presented as mean ± SEM. Statistical comparison was achieved using one-way ANOVA or the unpaired two-tailed *t*-test depending on the type of data set and as mentioned in the corresponding figure legend. Spearman correlation was used to analyze various parameters in clinical samples. *p* values ≤ 0.05 were considered to be significant as indicated: * = *p* < 0.05; ** = *p* < 0.01; *** = *p* < 0.001; **** = *p* < 0.0001; ns: non-significant.

## 3. Results

### 3.1. Characteristics of SGBS Organoids

**Figure 1:**
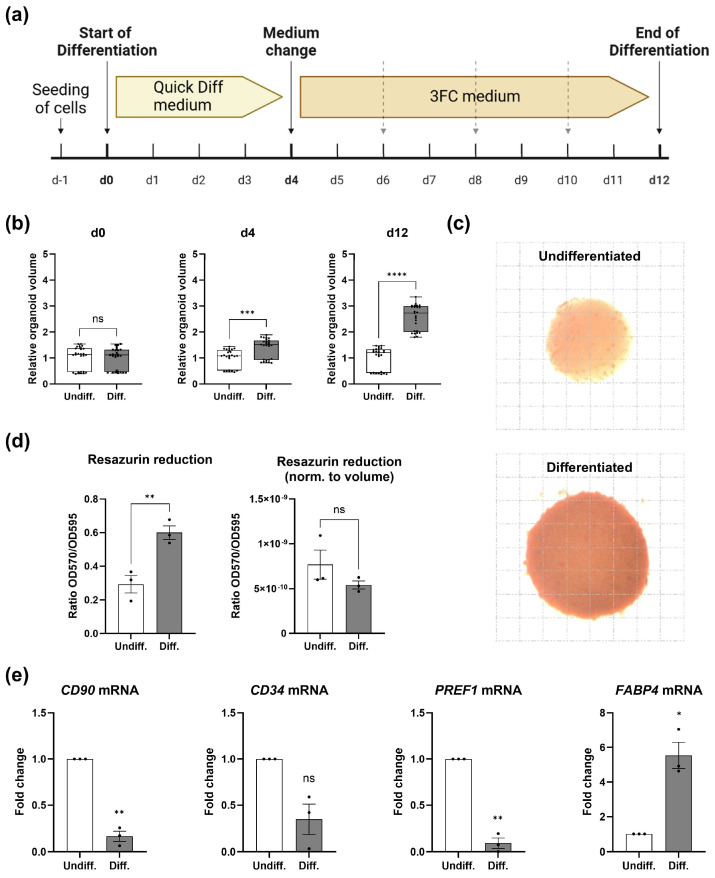
SGBS organoid model. (**a**) Overview of SGBS differentiation in low-attachment plates. After adipogenic induction (until d4), the medium was replaced every second or third day as indicated by dashed arrows. (**b**) Normalized organoid volume at selected time points. Individual organoids derived from n = 3 biological replicates were measured. Unpaired two-tailed *t*-test. (**c**) Oil Red-O staining of undifferentiated and differentiated organoids. Representative result of n = 3 experiments. Grid: 100 µm. (**d**) Resazurin reduction assay. Values are presented as mean ± SEM of n = 3 experiments. (**Left panel**): raw data; (**right panel**): normalization to organoid volume. The loss of a statistical difference between both groups using normalized data indicates that the observed effect (i.e., redox capacity) is due to cell intrinsic properties rather than an increased volume of differentiated organoids. Unpaired two-tailed *t*-test. (**e**) RT-qPCR analysis of stem cell- and pre-adipocyte markers of isolated nuclei derived from SGBS organoids. SGBS organoids were generated as outlined above followed by the isolation of nuclei to remove intracellular triglycerides that might interfere with gene expression analysis. Values are presented as mean ± SEM of n = 3 biological replicates. One-sample *t*-test. Spearman correlation was used to analyze various parameters in clinical samples. *p* values ≤ 0.05 were considered to be significant as indicated: * = *p* < 0.05; ** = *p* < 0.01; *** = *p* < 0.001; **** = *p* < 0.0001; ns: non-significant.

To establish a valid 3D cell culture model to recapitulate adipose tissue physiology, we used human Simpson–Golabi–Behmel Syndrome (SGBS) cells, which are known to express *UCP1* upon adipogenic differentiation in the presence of Rosiglitazone, among other browning factors [33]. SGBS cells were differentiated in 96-well low-attachment plates to induce organoid formation (Figure 1a). SGBS organoids increased in size over time (Figure 1b), accumulated intracellular lipids (Figure 1c) and showed a higher cellular redox capacity (Figure 1d) compared to undifferentiated counterparts. Next, nuclei were isolated (Supplementary Figure S1) and subjected to RT-qPCR analysis. As anticipated, the mRNA expression of stem cell- and pre-adipocyte markers such as *CD90* [45] and *PREF1* [46], respectively, decreased during adipogenic differentiation, whereas expression of the adipogenic marker *FABP4* [38] increased (Figure 1e). Taken together, the SGBS organoid model shows characteristic features of adipogenesis as observed in similar 3D cell culture systems [38].

### 3.2. snRNA-Seq Reveals Heterogeneity and Sub-Population Specific Responses to Hypoxia in SGBS Organoids

**Figure 2:**
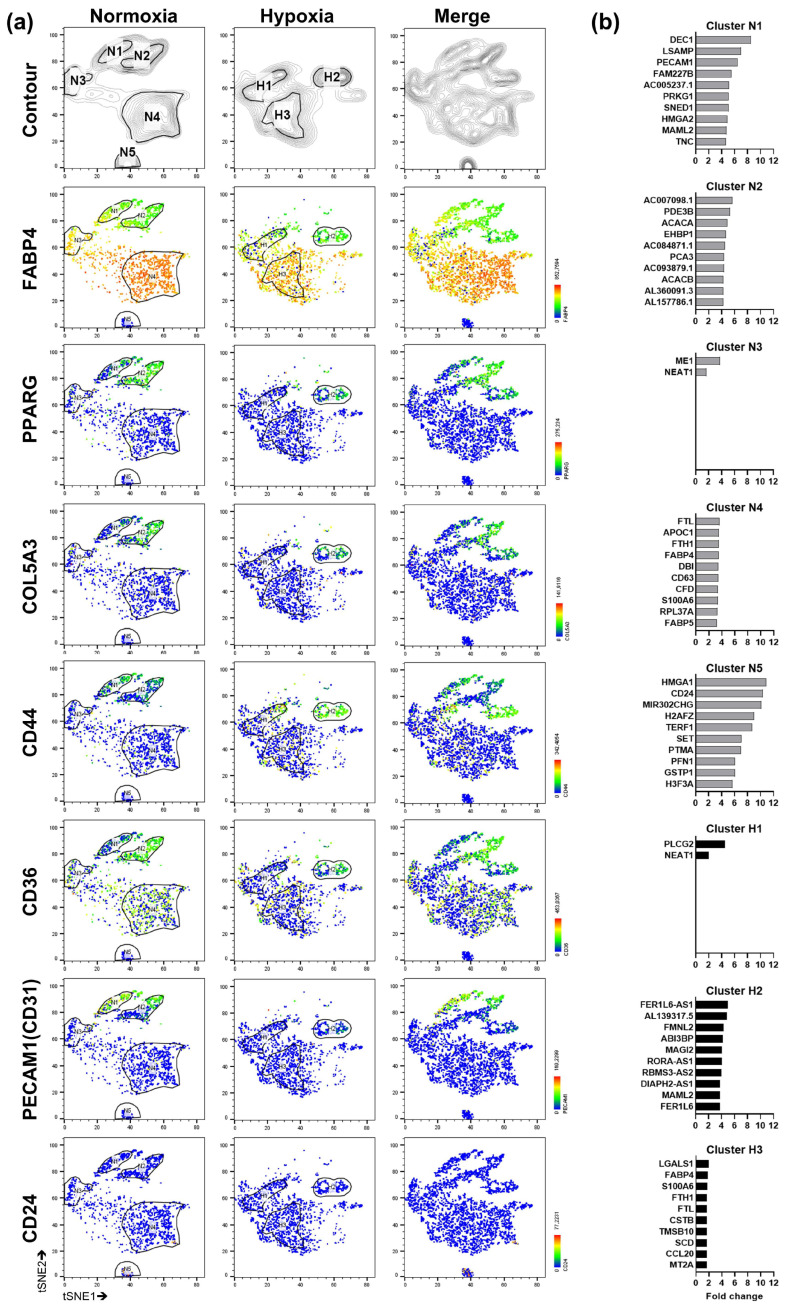
snRNA seq analysis of normoxic and hypoxic SGBS organoids (pool of n = 8–10 organoids each) (part 1). (**a**) t-Distributed stochastic neighbor embedding (t-SNE) plots. Clusters of nuclei found in SGBS organoids and heat maps showing the expression of various marker genes in normoxia (N), hypoxia (H) or both (Merge) as indicated. (**b**) Upregulated differential expressed genes (DEGs) in each cluster compared to all other clusters within the same experimental condition (i.e., either normoxia (N) or hypoxia (H)).

**Figure 3:**
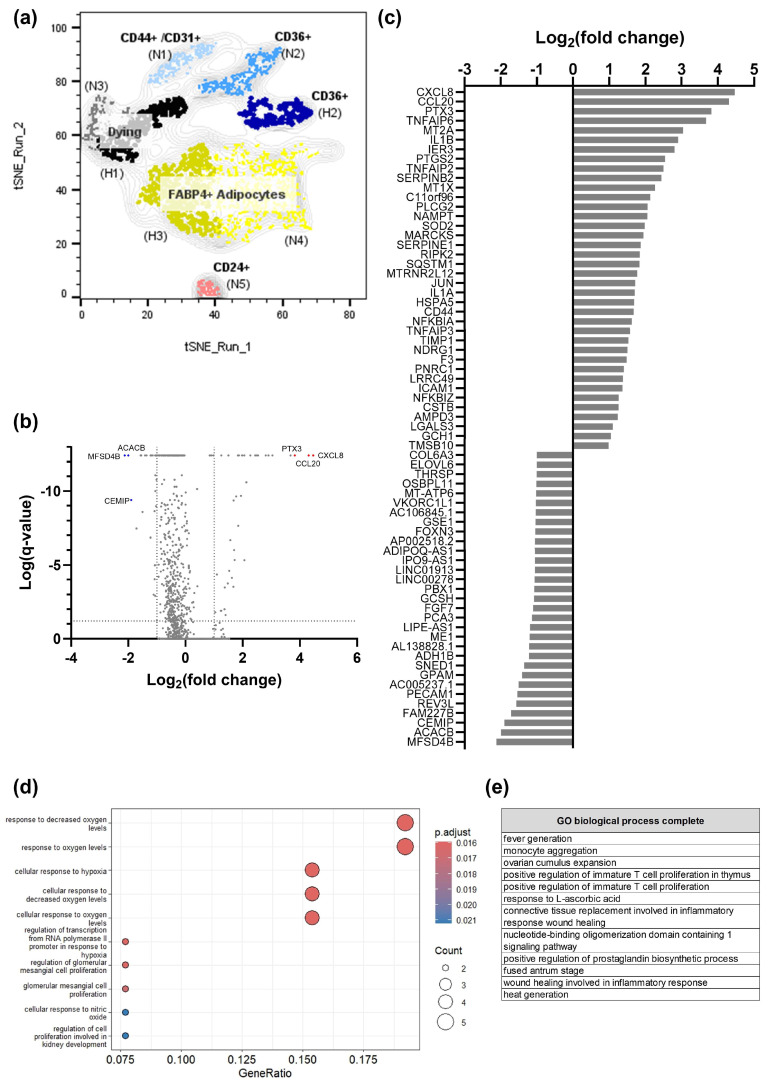
snRNA-seq analysis of normoxic (N) and hypoxic (H) SGBS organoids (pool of n = 8–10 organoids each) (part 2). (**a**) Cluster annotation according to expressed marker genes (see also Figure 2). Light colors represent normoxic conditions (cluster N1–N5), whereas dark colors highlight hypoxic conditions (cluster H1–H3). Frequencies: N1 10.7%, N2 16.2%, N3 6.75%, N4 41.2%, N5 4.65%, H1 15.5%, H2 13.0%, H3 27.4%. (**b**) Volcano plot of differential expressed genes (DEGs) in normoxia vs. hypoxia. Most highly regulated genes are indicated. (**c**) Bar chart of DEGs in normoxic and hypoxic SGBS organoids. (**d**) Gene Ontology (GO) enrichment analysis using the R package *clusterProfiler* of hypoxia-induced genes. (**e**) Gene Ontology (GO) enrichment analysis using the GO Resource of hypoxia-induced genes. Top enriched biological processes are presented (fold enrichment > 100).

To analyze SGBS organoids in more detail and to investigate the response to oxygen deprivation, SGBS organoids on d12 (Figure 1a) were exposed to hypoxia (3% O_2_) for 24 h or maintained at normoxia for comparison (Supplementary Figure S2a). Isolated nuclei stained predominantly positive for DAPI and Lamin A/C as determined by flow cytometry (Supplementary Figure S2b,c) and were subjected to snRNA-seq analysis (Supplementary Figure S3). In normoxia, five distinct clusters (i.e., sub-populations) were identified (designated as N1-5), whereas in hypoxia, only three clusters were detected (designated as H1-H3) (Figure 2a). Specific marker genes were highlighted according to published literature [43,44] (Figure 2a). Successful differentiation of SGBS cells into adipocytes was confirmed again by the appearance of a FABP4+ population, respectively (i.e., cluster N4 and H3) (Figure 2a). Next, the gene expression pattern of each cluster was compared to all clusters within the same experimental condition (either normoxia or hypoxia) and differential expressed genes (DEGs) were analyzed (Figure 2b). Subsequently, the upregulated gene sets of various clusters were matched (Supplementary Figure S3b,c). Based on DEG analysis and the expression of well-defined signature genes [43,44] (Figure 2), clusters were manually annotated (Figure 3a). Intriguingly, clusters N3 and H1 showed a transcriptional shutdown (Figure 2b), which is a hallmark of dying cells [47], whereas *NEAT1* (Figure 2b), a long-noncoding RNA associated with ferroptosis [48], was expressed. Further analysis of ferroptosis-associated genes (i.e., *GPX4*, *ACSL4*, *LPCAT3*, *NEAT1*, *SLC39A14* [48,49,50,51]) revealed a heterogeneous expression pattern among clusters and conditions (i.e., normoxia and hypoxia) (Supplementary Figure S4). In conclusion, these findings might imply ferroptosis to take place in SGBS organoids in general.

Next, the overall response of SGBS organoids to hypoxia was studied. DEG analysis demonstrated the induction of cytokines such as *CXCL8*, *CCL20* and *TNFA*, among others, in hypoxic organoids (Figure 3b,c; Supplementary Figures S5 and S6). Gene Ontology (GO) enrichment analysis of upregulated DEGs confirmed a transcriptional response to hypoxia (Figure 3d) and revealed a pro-inflammatory phenotype due to hypoxic exposure (Figure 3e). In addition, Reactome pathway analysis provided evidence of the presence of programmed cell death and a SASP expression pattern in hypoxic SGBS organoids (Supplementary Figure S7). Detailed comparison of normoxic and hypoxic FABP4+ adipocytes (cluster N4 vs. cluster H3) revealed again the induction of a pro-inflammatory gene expression pattern in hypoxia, while genes associated with ATP synthesis were downregulated in this cell type (Supplementary Figure S8). Taken together, these results demonstrate the induction of an inflammaging phenotype in hypoxic SGBS organoids.

### 3.3. ACSL4 Protein Expression Is Elevated in Differentiated SGBS Cells

**Figure 4:**
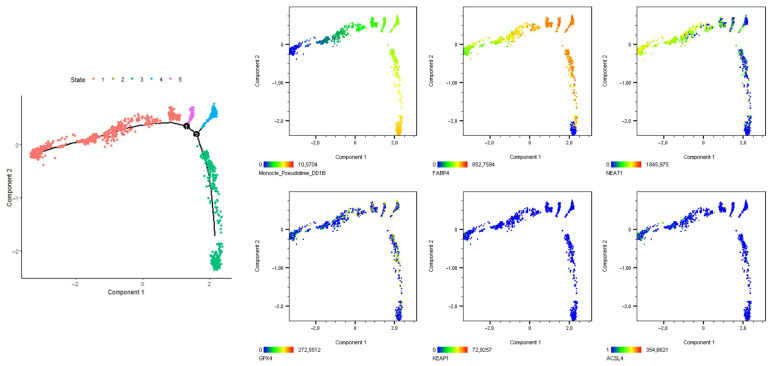
Analysis of ferroptosis-related factors in SGBS organoids. Pseudotime-trajectory analysis of normoxic SGBS organoids (corresponding to Figure 2 and Figure 3). (**Left panel**): identified stages by the Monocle algorithm. (**Right panel**), first row: Monocle pseudotime, FABP4 trajectory, NEAT1 trajectory; right panel, second row: GPX4 trajectory, KEAP1 trajectory, ACSL4 trajectory.

**Figure 5:**
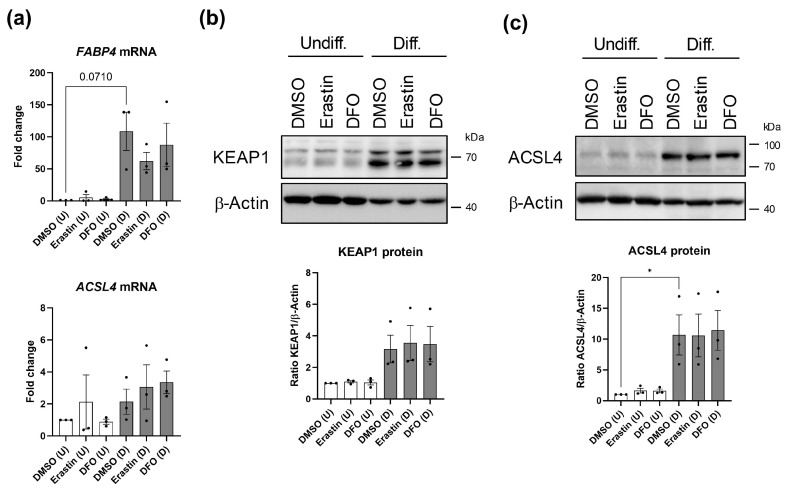
Analysis of ferroptosis-related factors in SGBS cells (2D cultures). (**a**) RT-qPCR analysis of undifferentiated (U) and differentiated (D) SGBS cells (2D cell cultures) treated with either DMSO, Erastin (10 µM, 24 h) or Desferrioxamine (DFO; 10 µM, 24 h). n = 3; one-way ANOVA with Dunn’s multiple comparison test. (**b**,**c**) Western blot analysis of undifferentiated (U) and differentiated (D) SGBS cells (2D cell cultures). SGBS cells were either differentiated into adipocytes or maintained in an undifferentiated state for comparison. Next, SGBS cultures of both conditions were treated with either DMSO, Erastin (10 µM, 24 h) or Desferrioxamine (DFO; 10 µM; 24 h). n = 3; one-way ANOVA with Dunn’s multiple comparison test. Spearman correlation was used to analyze various parameters in clinical samples. *p* values ≤ 0.05 were considered to be significant as indicated: * = *p* < 0.05.

Based on the snRNA seq data (Figure 2, Figure 3 and Supplementary Figure S4), it was hypothesized whether the capability to undergo ferroptosis might be related to adipogenesis. To solidify previous results, a pseudo-time trajectory analysis was computed using the snRNA seq data set derived from normoxic SGBS organoids (Figure 3a). Herein, *NEAT1* exhibited a clear trajectory inversely correlated with *FABP4* among differentiation states (Figure 4). However, ferroptosis-associated factors like *GPX4* or *ACSL4* showed a scattered appearance along the path, if any (i.e., *KEAP1*). To investigate this issue further and for convenience, we switched to canonical 2D cultures of undifferentiated and differentiated SGBS cells. In addition, SGBS cells were treated with the ferroptosis-inducer Erastin (10 µM) [35] or the ferroptosis-inhibitor Desferroxamine (10 µM) (DFO) [36] for 24 h as these compounds (or similar) were shown to increase or decrease ACSL4 in some models, respectively [52,53,54,55] (Figure 5). As anticipated, *FABP4* mRNA expression was elevated in differentiated cells compared to undifferentiated counterparts, whereas the *ACSL4* mRNA expression showed no clear pattern (Figure 5a) and *KEAP1* mRNA was hardly detectable. Intriguingly, KEAP1 protein levels tended to be elevated in differentiated SGBS cells, whereas ACSL4 protein was significantly induced during adipogenesis (Figure 5b,c). It is important to note that neither Erastin nor DFO treatment of SGBS cells showed a clear effect on KEAP1 and ACSL4 under the conditions used (Figure 5b,c). In conclusion, the presented results link ACSL4 abundance with adipogenesis.

### 3.4. ACSL4 Expression Is Induced by Brown-like Differentiation in Human WAT-Derived ASCs

**Figure 6:**
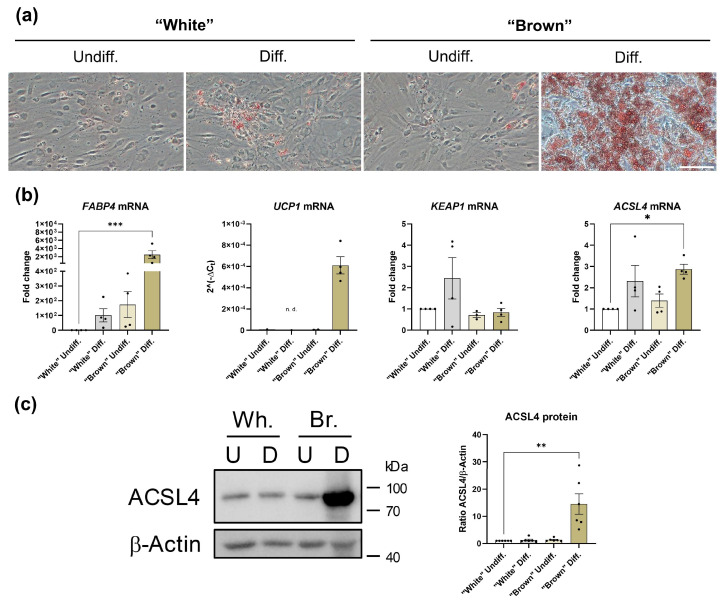
Differentiation of white adipose tissue (WAT)-derived human ASCs into either “white” (Wh.) or “brown-like” (Br.) adi-pocytes (2D cell cultures). (**a**) Microphotographs of undifferentiated (Undiff.) and differentiated (Diff.) ASCs. Two distinct protocols were applied. Representative result of n = 6 biological replicates. (**b**) RT-qPCR analysis of selected genes as indicated. n = 4. One-way ANOVA with Dunn’s multiple comparison test. n.d.: not detectable; (**c**) Western blot analysis. (**Left panel**): a representative ACSL4 Western blot of n = 6 biological replicates is shown. (**Right panel**): ACSL4 Western blot quantitation of n = 6 biological replicates. One-way ANOVA with Dunn’s multiple comparison test. Spearman correlation was used to analyze various parameters in clinical samples. *p* values ≤ 0.05 were considered to be significant as indicated: * = *p* < 0.05; ** = *p* < 0.01; *** = *p* < 0.001.

To investigate whether the capability to undergo ACSL4-mediated ferroptosis is a cell-specific feature of differentiated SGBS cells, human WAT-derived ASCs were differentiated either into “white-” or “brown-like” adipocytes in 2D cell cultures. For this purpose, Rosiglitazone, T3 and Cortisol were added (“brown”) or omitted (“white”) at the onset of adipogenesis. Successful differentiation was confirmed by intracellular lipid accumulation as evidenced by Oil Red O staining (Figure 6a). RT-qPCR analysis revealed a significant induction of *FABP4*, *UCP1* and *ACSL4* mRNA expression solely in brown-like adipocytes (Figure 6b). Western blot analysis confirmed a highly elevated ACSL4 abundance in this cell type (Figure 6c). In conclusion, these findings reveal that the capability to undergo ACSL4-mediated ferroptosis is acquired during brown-like differentiation of human ASCs.

### 3.5. Hypoxic Exposure of SGBS Organoids Downregulates ACSL4 Abundance but Promotes Oxidative Stress, Cellular Senescence, and Pro-Inflammatory Cytokine Release

**Figure 7:**
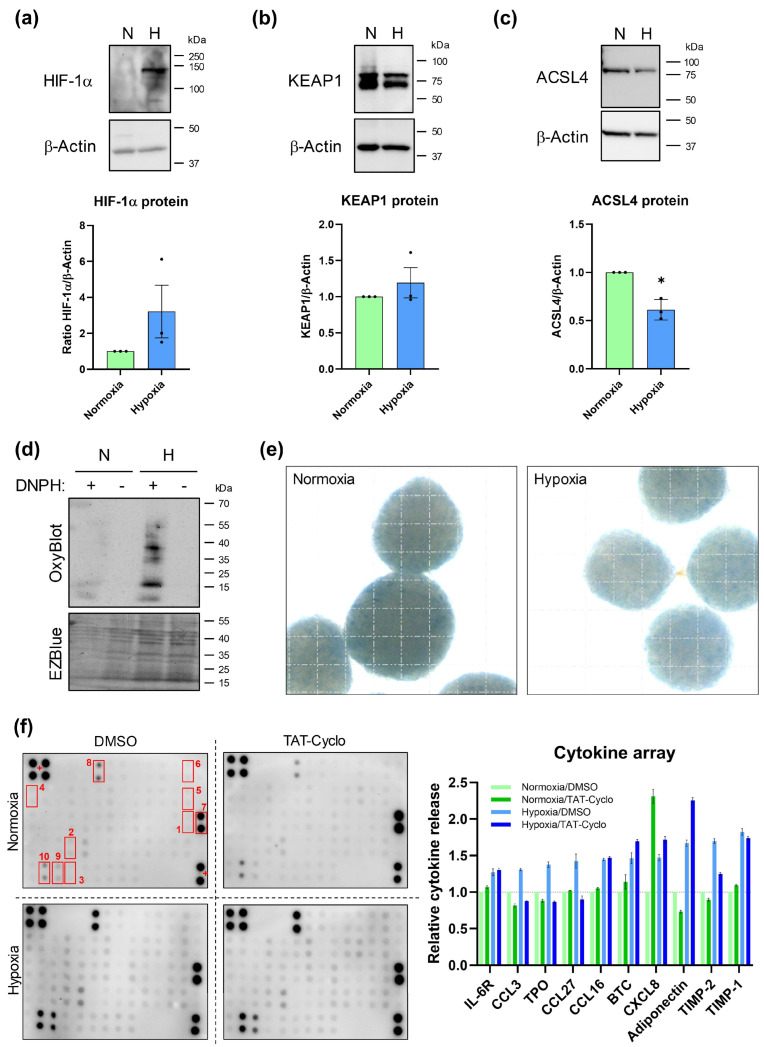
Effects of hypoxia in SGBS organoids. (**a**–**c**) Western blot analysis of normoxic (N) and hypoxic (H) SGBS organoids. n = 3; one-sample *t* test. (**d**) Analysis of protein oxidation by OxyBlot. A representative result of n = 3 independent experiments is shown. The DNPH substrate was added (+) or omitted (−) to confirm signal specificity. PVDF membrane was counterstained with EZBlue. (**e**) SA β-Gal staining of SGBS organoids. Grid: 100 µm. (**f**) Cytokine array. Cell culture supernatants of normoxic and hypoxic SGBS organoids of n = 3 experiments were pooled and subjected to cytokine measurement. TAT-Cyclo was used to inhibit HIF-1α. Top ten hypoxia-induced cytokines are indicated: (1) IL-6R, (2) CCL3, (3) TPO, (4) CCL27, (5) CCL16, (6) BTC, (7) CXCL8, (8) Adiponectin, (9) TIMP-2 and (10) TIMP-1. (+) Array positive control. Spearman correlation was used to analyze various parameters in clinical samples. *p* values ≤ 0.05 were considered to be significant as indicated: * = *p* < 0.05.

Next, we aimed to study the effect of hypoxia on ferroptosis-related factors. As presented in Figure 7a–c, hypoxia consistently downregulated ACSL4 on the protein level, whereas KEAP1 was not significantly affected. As anticipated by the snRNA seq data (Figure 3), hypoxic SGBS organoids showed elevated oxidative stress (Figure 7d), higher senescence-associated β-Galactosidase activity (Figure 7e and Supplementary Figure S9) and increased pro-inflammatory cytokine release (Figure 7f). Treatment of SGBS organoids with the HIF-1α inhibitor TAT-Cyclo-CLLFVY (TAT-Cyclo) [37] partly abrogated the induction of certain cytokines but increased others (Figure 7f), which might imply a counter-regulatory effect of HIF-2α and/or HIF-3α subunits under these conditions. In summary, the data demonstrate opposite effects of hypoxia on ACSL4-dependent ferroptosis-capacity (inhibition) and senescence-related phenotypes (promoting), thus suggesting a relationship.

### 3.6. ACSL4 and UCP1 mRNA Expression Correlates in EAT

**Figure 8:**
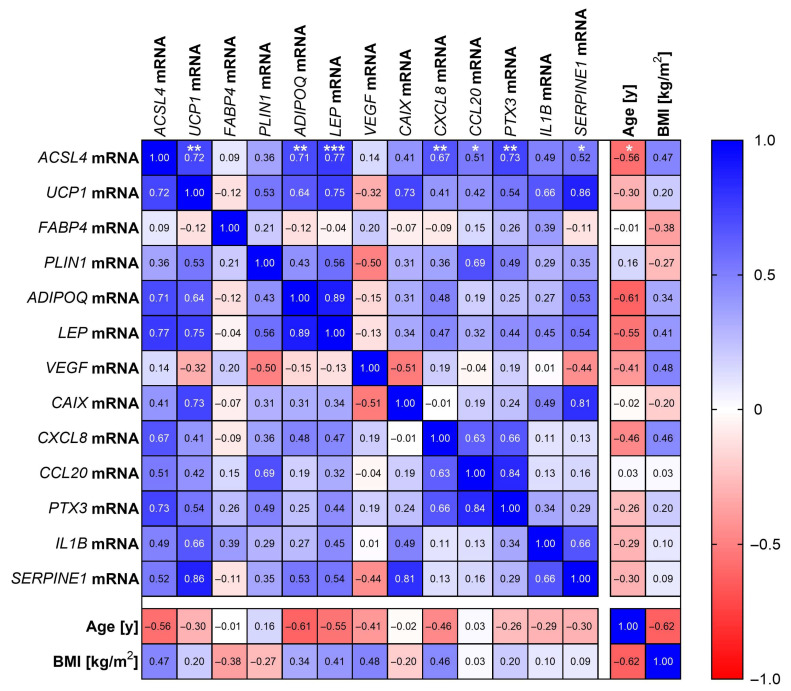
Spearman correlation matrix of gene expression measured in EAT (whole tissue; n = 16) as indicated. Correlation coefficients (r) are given. Statistical significance is only shown for ACSL4 mRNA for clarity. Spearman correlation was used to analyze various parameters in clinical samples. *p* values ≤ 0.05 were considered to be significant as indicated: * = *p* < 0.05; ** = *p* < 0.01; *** = *p* < 0.001.

Because our results clearly show the upregulation of ACSL4 in brown-like adipogenesis (Figure 6) and a downregulation by hypoxia (Figure 7), we asked whether there is an association between these factors in human brown adipose tissue samples. Therefore, we analyzed EAT samples (whole tissue; n = 16) obtained from patients undergoing cardiac surgery by RT-qPCR and Western blotting. As EAT is known to undergo brown-to-white trans-differentiation during aging [15] and is impaired by obesity [56], we also correlated the expression of all adipose tissue, hypoxia and inflammatory markers to age and BMI, respectively. The analysis revealed a correlation of *ACSL4* and *UCP1* mRNA, thus supporting our previous findings that *ACSL4* is linked to brown-like adipogenesis (Figure 8). Intriguingly, *ACSL4* mRNA correlated with hypoxia-inducible genes that were also highly expressed in oxygen-deprived SGBS organoids (e.g., *CXCL8*, *CCL20*, *PTX3*; Figure 3; Figure 8). However, ACSL4 mRNA and protein levels appeared to be inversely correlated (Supplementary Figure S10). In conclusion, these findings support a link between ACSL4 and brown-like adipocytes and provide further evidence of the regulation of ACSL4 by hypoxia.

## 4. Discussion

In the present study, we aimed to elucidate the effects of hypoxia in SGBS organoids in an unbiased manner. SGBS organoids showed similar characteristics as observed in other scaffold-free 3D cell culture systems used by various groups [38,57,58,59], such as an increase in size over time, the expression of specific marker genes and the accumulation of lipids. The storage of triglycerides is a hallmark of adipocytes [11] but renders downstream analysis on a single-cell basis complicated as lipids might interfere with detection systems [47]. To overcome this limitation, we applied single-nucleus RNA sequencing (snRNA seq) to elucidate the composition of SGBS organoids and their response to hypoxia in detail. For this purpose and not to miss anything, no filtering was applied in the snRNA seq data. Unsupervised clustering revealed a different number of populations in normoxia (5 cluster) and hypoxia (3 cluster), thereby suggesting the disappearance of cell types probably due to cell death in hypoxia. Indeed, analysis of differential expressed genes among clusters within the same condition highlighted one population each of reduced transcriptional activity, which is indicative of dead or dying cells [47]. Intriguingly, the same clusters expressed NEAT1, a long noncoding RNA associated with ferroptosis [48], thus pointing towards a possible mechanism of cell death. The fact that *NEAT1* expression was evident in one cluster of transcriptional shutdown in normoxia and hypoxia, respectively, implies ferroptosis takes place in general in SGBS organoids. Of note, this finding does not exclude any other form of programmed cell death nor necrosis in this model. Moreover, snRNA seq revealed the induction of a pro-inflammatory SASP expression pattern in hypoxic SGBS organoids, which was confirmed by elevated oxidative stress, increased senescence-associated β-Galactosidase positivity, and pro-inflammatory cytokine secretion. Therefore, these findings prompted us to investigate the relationship between hypoxia and ferroptosis-related factors in adipocytes.

Ferroptosis is an iron-dependent form of programmed cell death involving the peroxidation of lipids [25]. As ACSL4 plays a key role in canonical ferroptosis execution [25], we focused on the capability of pre-adipocytes to acquire this function. Of note, ACSL4 expression alone is not indicative for ferroptosis per se as other hallmarks (e.g., mitochondrial alterations) need to be considered [28]. In addition, an ACSL4-independent form of ferroptosis also might exist [60,61,62]. Our results using SGBS cells and WAT-derived ASCs revealed that *ACSL4* expression is induced solely during brown-like adipogenesis by addition of Rosiglitazone, Cortisol and T3 to the differentiation medium, thereby providing a causal relationship. In line with this observation, ACSL4 and UCP1 mRNA expression were significantly correlated in human EAT, a unique fat depot with brown-like characteristics [15]. Rosiglitazone activates PPARγ, a key transcription factor in adipogenesis and browning [13,14], thus suggesting PPARγ (or its target(s)) plays a major role in ferroptosis regulation. In theory, this raises the possibility that ferroptosis might be influenced in adipose tissue systemically by PPARγ agonists or antagonists depending on the desired outcome. Recently, Wang et al. demonstrated that adipocyte-specific overexpression of *ACSL4* improves ferroptotic signaling and mitigates obesity in vivo [29]. A possible role of ferroptosis in obesity was also reported by others [63]. Taken together, exploring the regulation of ACSL4 might hold promise to find novel avenues to combat obesity and to promote healthy aging as both conditions are tightly linked [64,65].

While ACSL4 mRNA expression was not highly affected in SGBS cells by various conditions, ACSL4 protein was significantly upregulated upon differentiation. In agreement, brown-like adipogenesis of WAT-derived ASCs lead to an unequal induction of ACSL4 mRNA and protein levels. Our analysis of EAT samples revealed that ACSL4 mRNA and protein levels tend to be inversely correlated. These findings imply a strong post-transcriptional and/or post-translational mechanism of ACSL4 regulation in human brown-like adipocytes.

In our model, we observed an inhibitory effect of hypoxia on ACSL4. To answer the question how hypoxia might inhibit ACSL4, gene set enrichment analysis of hypoxia-induced genes in SGBS organoids was performed using the FerrDb V2 database [66]. This analysis identified five candidate genes (i.e., *SQSTM1*, *JUN*, *HSPA5*, *CD44*, *GCH1*) as putative ferroptosis suppressors among the selected data set. However, *SQSTM1* and *JUN* are able to inhibit ferroptosis in a cell specific-context [67,68,69,70]. *HSPA5*, encoding heat shock 70 kDa protein 5, was shown to protect Glutathione peroxidase 4 (GPX4) from degradation, an enzyme required to reduce lipid peroxidation, thereby counteracting ferroptosis [71]. Moreover, GTP cyclohydrolase-1 (GCH1) is involved in lipid remodeling [72], whereas the surface protein CD44 is a known modulator of cell death and regulates iron metabolism [73]. As iron plays a major role in ferroptosis and HIF signaling [20,28,29], a mutual regulation of both processes is obvious. However, several reports describe opposite roles of hypoxia on ferroptosis [49,74,75]. This divergence might be attributed to a tissue-specific expression pattern of HIF subunits in addition to severity and duration of hypoxia applied, as different HIF isoforms respond to various conditions [76]. Further studies are needed to elucidate the interplay between these pathways regarding the inhibitory effect of hypoxia on ACSL4, especially in various AT depots and in the context of pathophysiological conditions such as obesity and aging.

## 5. Conclusions

**Figure 9:**
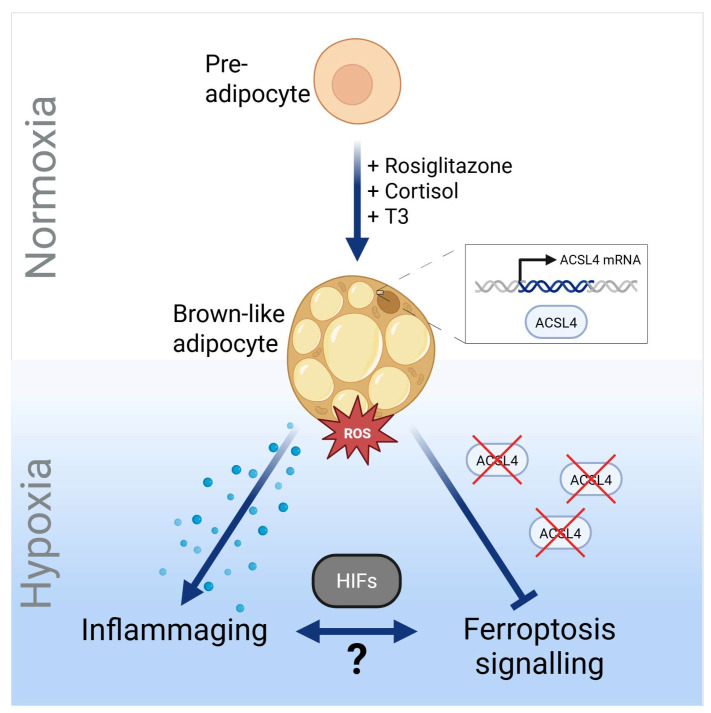
Working model. (Created in BioRender; https://BioRender.com/2yzl2l3 (accessed on 9 August 2025)).

Our study reveals that the capability to undergo ACSL4-mediated ferroptosis of adipocytes is linked to brown-like adipogenesis. This implies the possibility to modulate ferroptosis by soluble factors affecting browning, putatively by PPARγ agonists and antagonists in adipose tissue. Our results demonstrate an inhibitory effect of hypoxia on ACSL4 that is accompanied by inflammaging (Figure 9). Whether there is a bi-directional relationship between both processes needs to be determined by future studies.

## Data Availability

The data sets generated during and/or analyzed during the current study are available from the corresponding author on reasonable request.

## Supplementary Materials

The following supporting information can be downloaded at https://www.mdpi.com/article/10.3390/cells14161247/s1: Supplementary Table S1: Primer sequences used for RT-qPCR analysis; Supplementary Figure S1: Nuclei isolation from undifferentiated and differentiated SGBS organoids; Supplementary Figure S2: Characterization of isolated nuclei prior snRNA seq; Supplementary Figure S3: snRNA seq data analysis from normoxic and hypoxic SGBS organoids (part A); Supplementary Figure S4: snRNA seq data analysis from normoxic and hypoxic SGBS organoids (part B); Supplementary Figure S5: snRNA seq data analysis from normoxic and hypoxic SGBS organoids (part C); Supplementary Figure S6: snRNA seq data analysis from normoxic and hypoxic SGBS organoids (part D); Supplementary Figure S7: snRNA seq data analysis from normoxic and hypoxic SGBS organoids (part E); Supplementary Figure S8: snRNA seq data analysis from normoxic and hypoxic SGBS organoids (part F); Supplementary Figure S9: SA β-Gal staining of cryo-sections derived from SGBS organoids; Supplementary Figure S10: Analysis of EAT samples derived from n = 16 patients undergoing cardiac surgery.

## Abbreviations

The following abbreviations are frequently used in this manuscript:

ACSL4	Acyl-CoA synthetase long-chain family member 4
ASCs	Adipogenic stem/progenitor cells
AT	Adipose tissue
BAT	Brown adipose tissue
EAT	Epicardial adipose tissue
SGBS	Simpson–Golabi–Behmel Syndrome
snRNA seq	Single-nucleus RNA sequencing
WAT	White adipose tissue

## Appendix A

**Table A1:** Patients undergoing cardiac surgery and donating epicardial adipose tissue. m: male; f: female; BMI: body mass index.

Patient #	Sex (m/f)	Age (y)	BMI (kg/m^2^)
1	m	75.75	24.82
2	m	83.43	26.67
3	f	78.54	23.71
4	m	73.14	25.26
5	m	67.04	27.99
6	m	76.14	27.18
7	f	74.88	18.75
8	f	54.36	35.55
9	m	60.66	40.00
10	m	69.10	29.53
11	m	76.98	22.38
12	m	56.19	24.48
13	m	82.68	23.75
14	f	78.83	19.44
15	f	54.27	29.56
16	m	75.21	24.20
