# Supplementary material for: The Capability to Undergo ACSL4-Mediated Ferroptosis Is Acquired During Brown-like Adipogenesis and Affected by Hypoxia
Source: Cells. 2025 Aug 13;14(16):1247. doi: 10.3390/cells14161247 (PMC12384742; doi:10.3390/cells14161247)
Supplement: Supplementary file 1 [file cells-14-01247-s001.zip › cells-3790546-supplementary.pdf]

Supplementary Figure legends

Supplementary Figure S1: Nuclei isolation from (a) undifferentiated and (b) differentiated SGBS organoids. A representative result of n=3 experiments is shown. (c) Average size and DAPI staining intensity of isolated nuclei derived from undifferentiated and differentiated SGBS organoids. n=3; unpaired t-test;

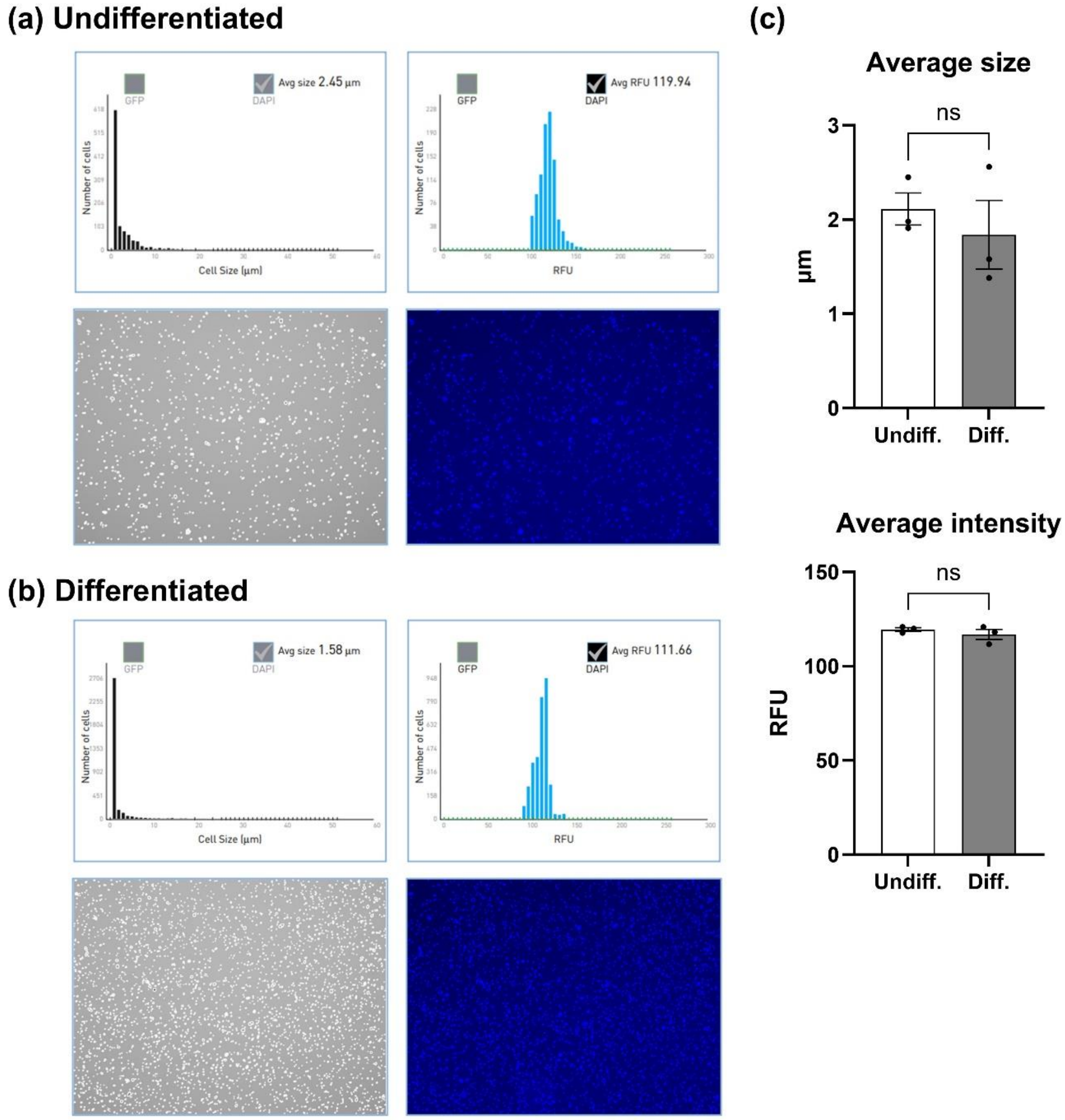

Supplementary Figure S2: Characterization of isolated nuclei prior snRNA seq. (a) Representative staining of SGBS organoids with the hypoxia marker Image-iT ™ Green Hypoxia Reagent (Invitrogen/ThermoScientific). (b) and (c): flow cytometry data of nuclei derived from normoxic and hypoxic SGBS organoids. n=8-10 organoids per condition were used;

(a)

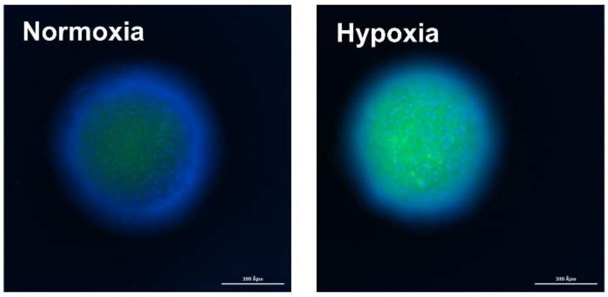

(b) Normoxia

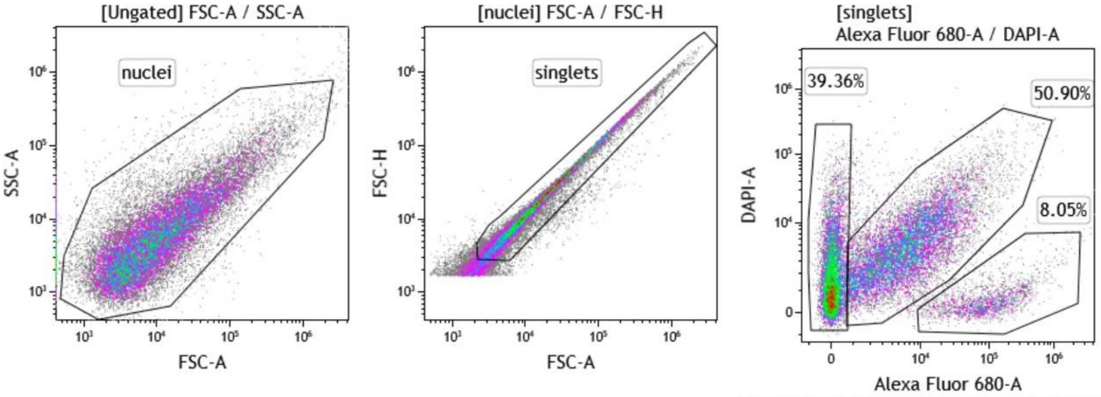

Gate	%Gated
DAPI- Lamin A/C+	8.05
DAPI+ Lamin A/C+	50.90
Lamin A/C-	39.36

(c) Hypoxia

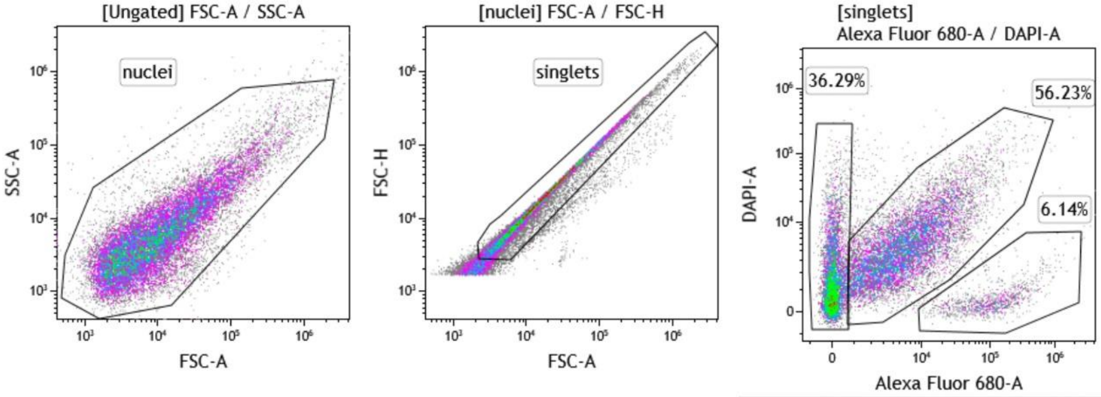

Gate	%Gated
DAPI- Lamin A/C+	6.14
DAPI+ Lamin A/C+	56.23
Lamin A/C-	36.29

Supplementary Figure S3: snRNA seq data analysis from normoxic and hypoxic SGBS organoids (part A). (a) Knee-plot. (b) and (c): comparison of DEG between clusters as indicated.

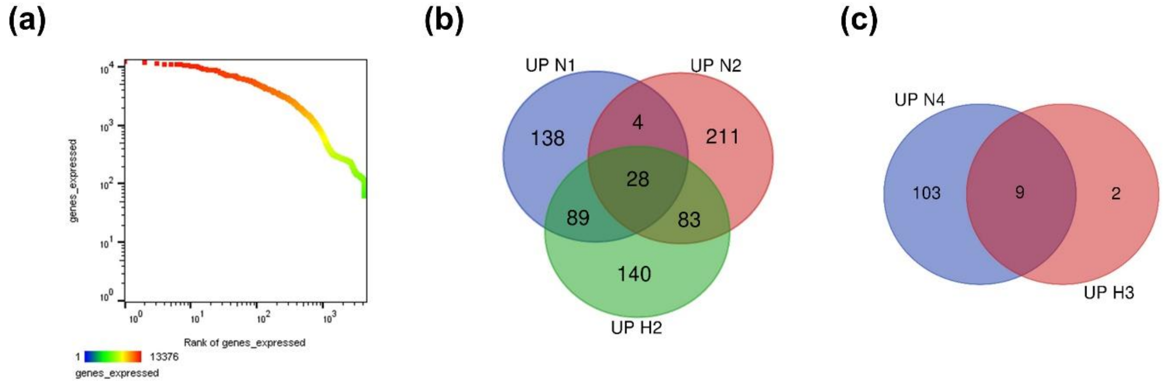

Supplementary Figure S4: snRNA seq data analysis from normoxic and hypoxic SGBS organoids (part B). Expression of various ferroptosis markers in different clusters is shown as indicated.

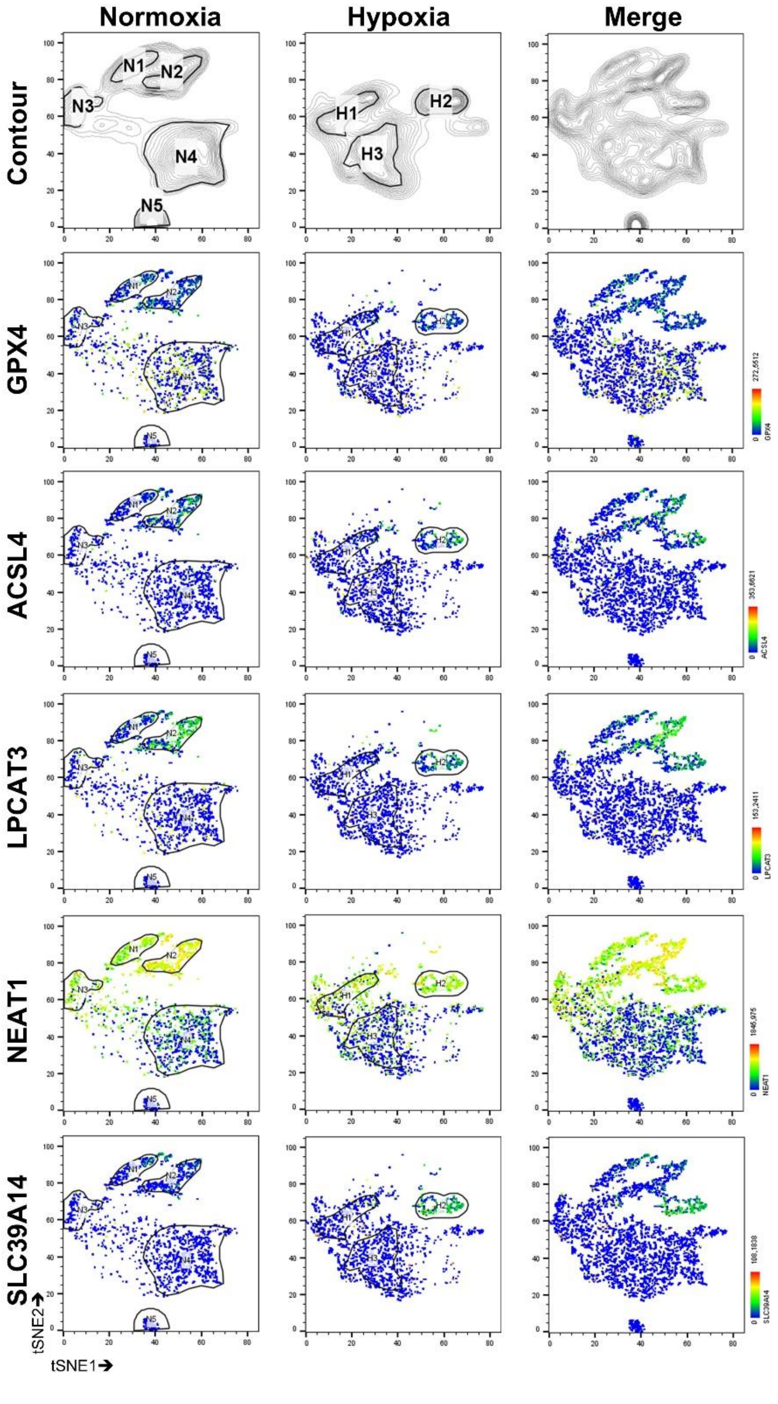

Supplementary Figure S5: snRNA seq data analysis from normoxic and hypoxic SGBS organoids (part C). Expression of various pro-inflammatory cytokines in different clusters is shown as indicated.

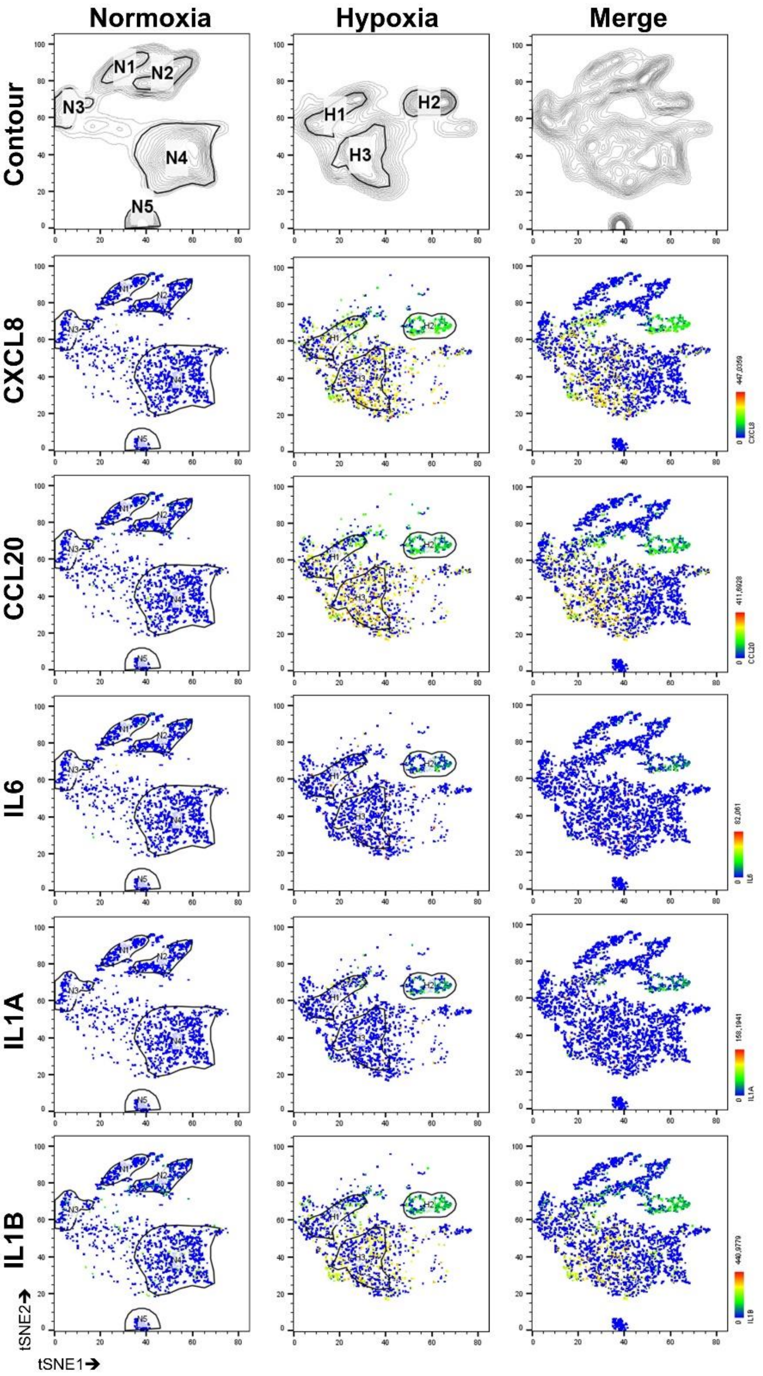

Supplementary Figure S6: snRNA seq data analysis from normoxic and hypoxic SGBS organoids (part D). Heat map of (a) normoxic and (b) hypoxic data set.

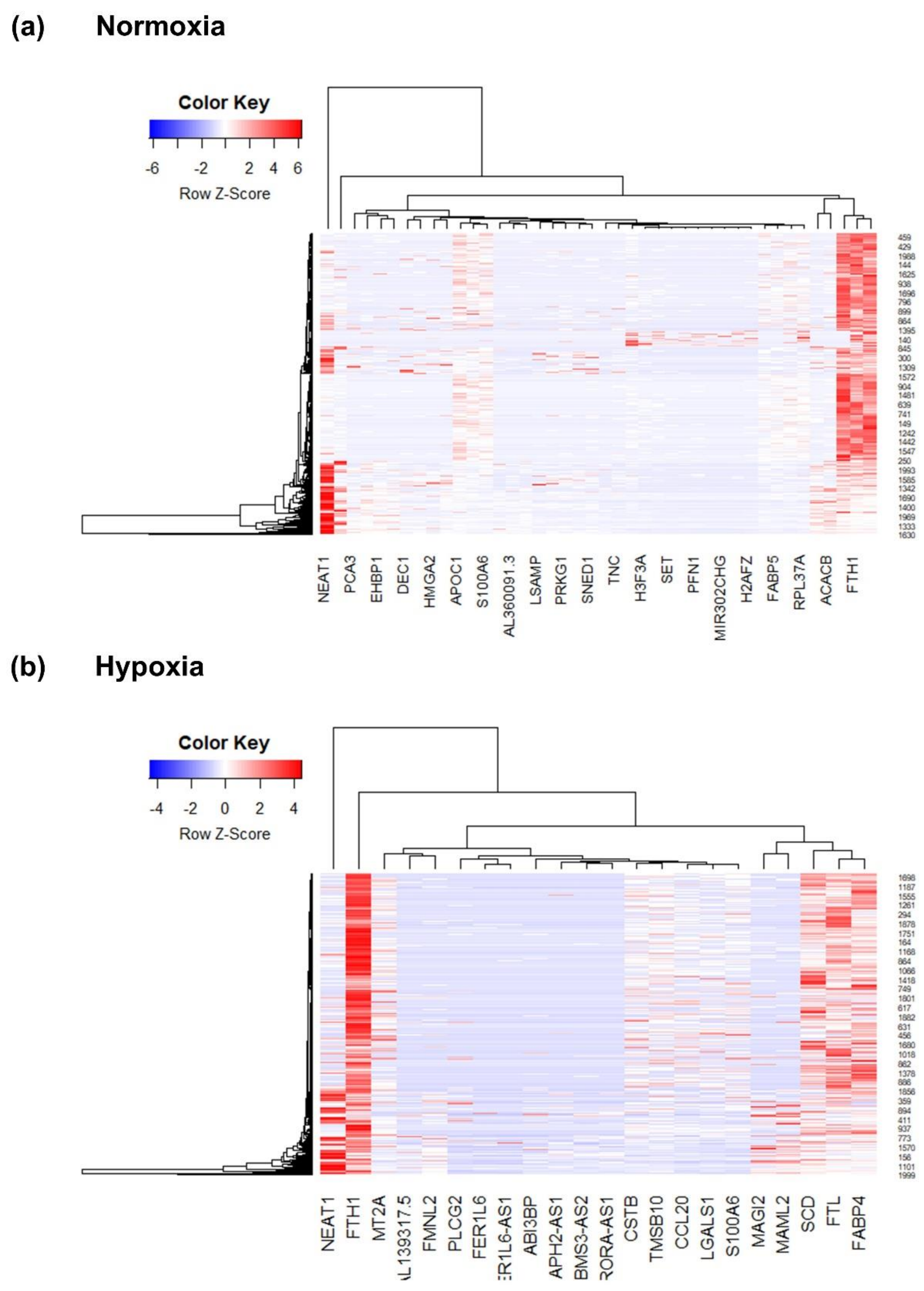

Supplementary Figure S7: snRNA seq data analysis from normoxic and hypoxic SGBS organoids (part E). Reactome pathway analysis of hypoxia induced genes.

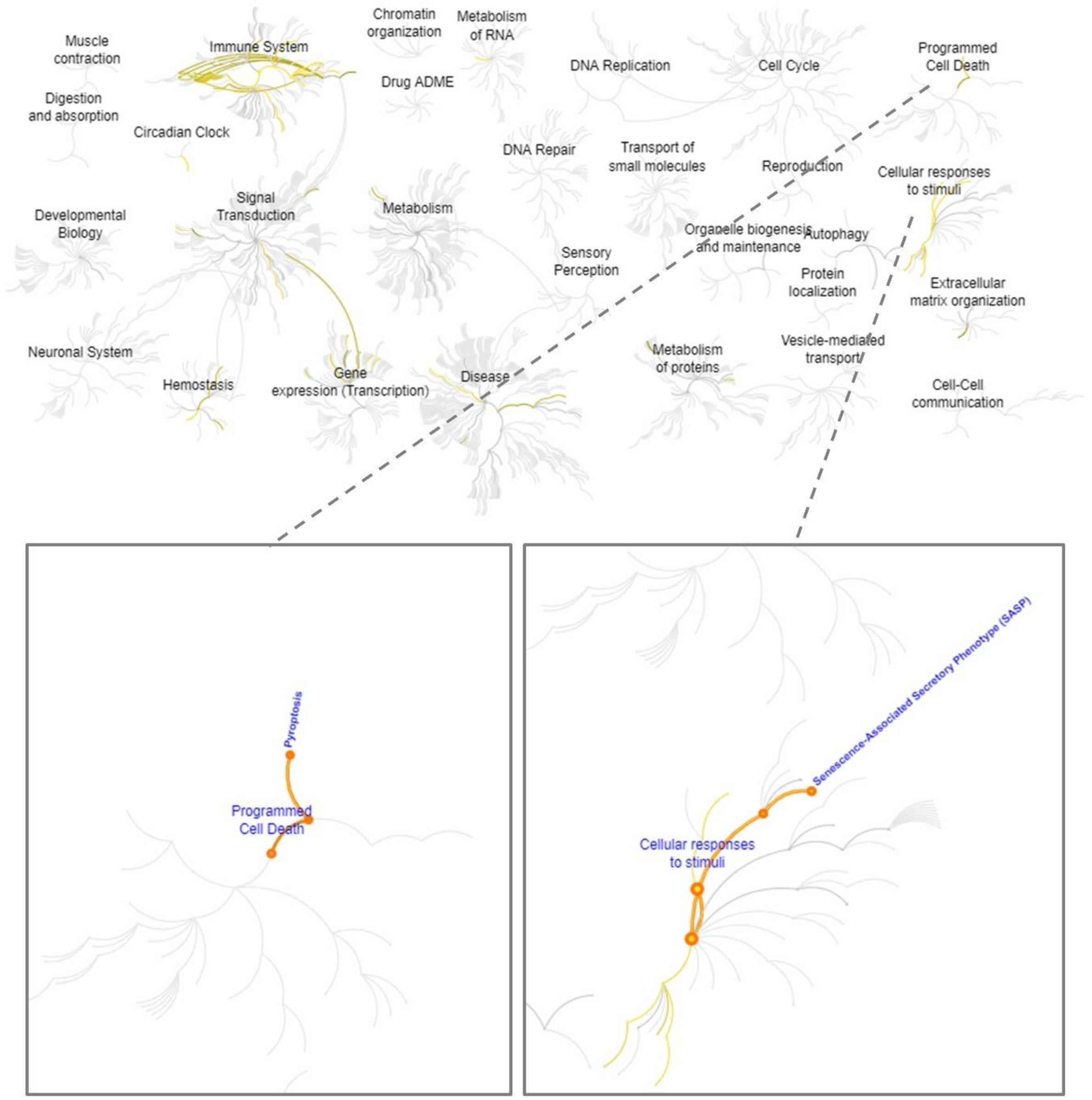

Supplementary Figure S8: snRNA seq data analysis from normoxic and hypoxic SGBS organoids (part F). (a) DEG in normoxic and hypoxic adipocytes (cluster N4 vs. H3). (b) Gene ontology of up- and down-regulated genes (normoxia vs. hypoxia).

(a)

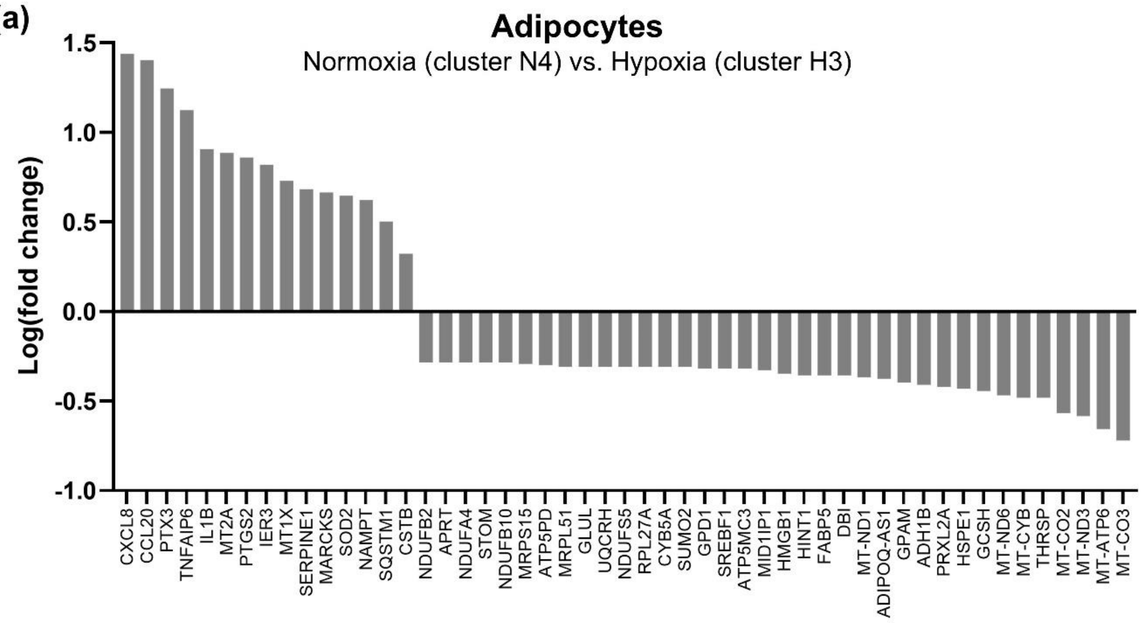

(b)

	GO biological process complete	Fold Enrichment	FDR
Up-regulated Genes	ovarian cumulus expansion	> 100	3.45E-03
	positive regulation of prostaglandin biosynthetic process	> 100	4.61E-03
	fused antrum stage	> 100	4.45E-03
	positive regulation of fever generation	> 100	5.48E-03
	cellular response to erythropoietin	> 100	5.32E-03
	response to erythropoietin	> 100	5.17E-03
	regulation of cell adhesion molecule production	> 100	6.34E-03
	positive regulation of unsaturated fatty acid biosynthetic process	> 100	6.18E-03
	positive regulation of heat generation	> 100	6.02E-03
	regulation of fever generation	> 100	7.20E-03
Down-regulated Genes	glycerol-3-phosphate metabolic process	> 100	2.51E-02
	alditol phosphate metabolic process	98.06	3.63E-02
	mitochondrial electron transport, NADH to ubiquinone	89.53	7.65E-10
	mitochondrial electron transport, ubiquinol to cytochrome c	84.05	4.80E-02
	mitochondrial electron transport, cytochrome c to oxygen	73.54	2.02E-03
	proton motive force-driven mitochondrial ATP synthesis	73.54	1.16E-10
	aerobic electron transport chain	73.54	3.80E-15
	proton motive force-driven ATP synthesis	72.54	4.82E-12
	mitochondrial ATP synthesis coupled electron transport	69.59	6.29E-15
	ATP synthesis coupled electron transport	69.59	5.59E-15

Supplementary Figure S9: SA β-Gal staining of cryo-sections derived from SGBS organoids. A representative result of n=3 experiments is shown. Scale bar: 100 μm.

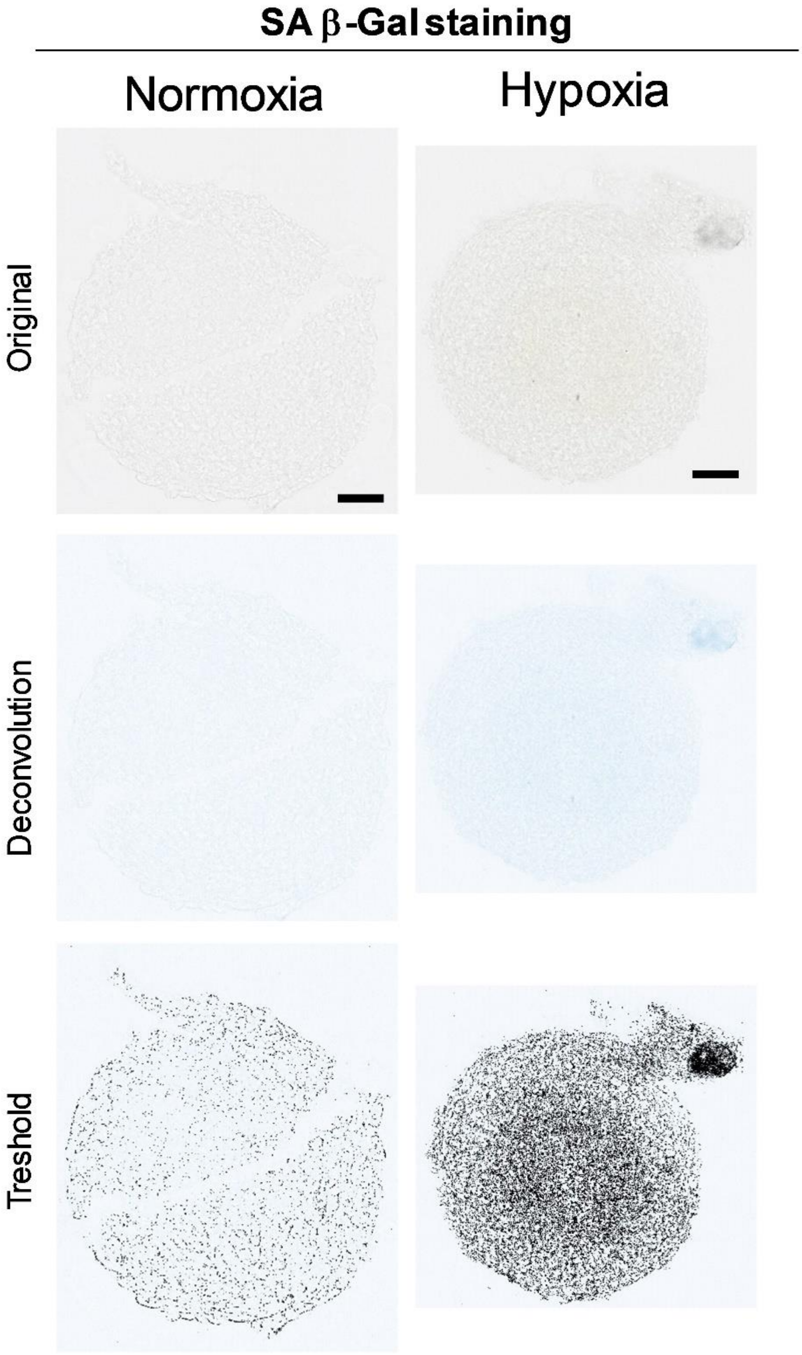

Supplementary Figure S10: Analysis of EAT samples derived from n=16 patients undergoing cardiac surgery. Patient characteristics are provided in **Table A1**. (a) Western blot analysis of EAT protein samples derived from patients 1-8. β-Actin was used for normalization. (b) Western blot analysis of EAT protein samples derived from patients 9-16. β-Actin was used for normalization. (c) Spearman correlation matrix corresponding to (a) as indicated. (d) Spearman correlation matrix corresponding to (b) as indicated. Only patients #11, 13, 15 and 16 were included in the analysis due to technical limitations and low sample amount. (e) Spearman correlation of ACSL4 mRNA and protein (normalized) of the pooled data set from (a) and (b).

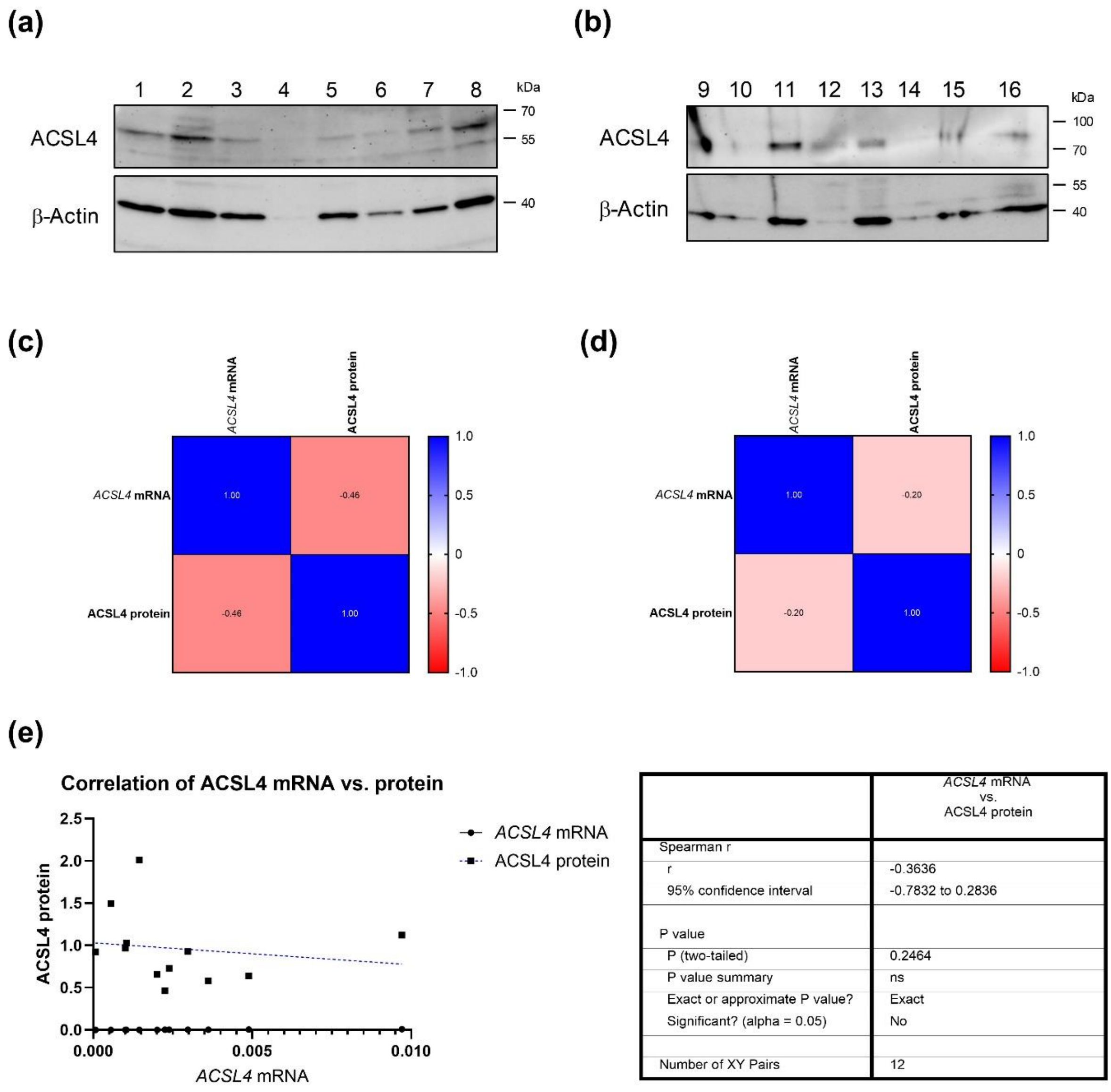

	ACSL4 mRNA vs. ACSL4 protein
Spearman r	
r	-0.3636
95% confidence interval	-0.7832 to 0.2836
P value	
P (two-tailed)	0.2464
P value summary	ns
Exact or approximate P value?	Exact
Significant? (alpha = 0.05)	No
Number of XY Pairs	12

Supplementary Table 1 - Primer sequences

Primer sequences used for RT-qPCR (human)

Name	Sequence (5´- 3´)
h_ACSL4_for	CGGTTCCTTTTTGCGAGCTT
h_ACSL4_rev	ACACGAATATCTTCTGTGATTTCAA
h_ADIPOQ_for	TGACCAGGAAACCACGACTC
h_ADIPOQ_rev	CCCTTAGGACCAATAAGACCTGG
h_CAIX_for	CCTCTGACTTCAGCCGCTAC
h_CAIX_rev	GTGTCAGAGAGGGTGTGGAG
h_CCL20_for	TTGTCTGTGTGCGCAAATCC
h_CCL20_rev	CTCCAACCCCAGCAAGGTTC
h_CD34_for	TGATTGCACTGGTCACCTCG
h_CD34_rev	TAAGGGTCTTCGCCCAGCC
h_CD90_for	CGGAAGACCCCAGTCCAGAT
h_CD90_rev	GGGAGACCTGCAAGACTGTT
h_CXCL8_for	AGAGAGCTCTGTCTGGACCC
h_CXCL8_rev	TTCTCAGCCCTCTTCAAAAACT
h_EPAS1_for	CCTTCCGACTCCCAGCATTC
h_EPAS1_rev	GGCTGTCAGACCCGAAAAGA
h_FABP4_for	AAACTGGTGGTGGAATGCGT
h_FABP4_rev	GCGAACTTCAGTCCAGGTCA
h_GAPDH_for	GAGTCAACGGATTTGGTCGT
h_GAPDH_rev	GACAAGCTTCCCGTTCTCAG
h_HIF1A_for	TGACAAGCCACCTGAGGAGA
h_HIF1A_rev	GGAAAGGCAAGTCCAGAGGT
h_IL1A_for	CAGCCAGAGAGGGAGTCATTT
h_IL1A_rev	TGTCTGGAACTTTGGCCATCTT
h_IL1B_for	TCGCCAGTGAAATGATGGCT
h_IL1B_rev	GGTCGGAGATTCGTAGCTGG

h_KEAP1_for	CCAACCGACAACCAAGACCC
h_KEAP1_rev	CTGCATGGGGTTCCAGAAGA
h_LEP_for	AATGCATTGGGGAACCCTGT
h_LEP_rev	AGGAGACTGACTGCGTGTGT
h_PLIN1_for	AGGGAAGAAGTTGAAGCTTGAGG
h_PLIN1_rev	TTCTGGAAGCATTCGCAGGT
h_PPARg2_for	AGAAAGCGATTCCTTCACTGAT
h_PPARg2_rev	AGAATGGCATCTCTGTGTCAAC
h_Pref1_for	CACGGACTCTGTGGAGAACC
h_Pref1_rev	GCAGGCCCGAACATCTCTAT
h_PTX3_for	GCTCTCTGGTCTGCAGTGTT
h_PTX3_rev	CTTGTCCCATTCCGAGTGCT
h_SERPINE1_for	AGAGCGCTGTCAAGAAGACC
h_SERPINE1_rev	AGTTCTCAGAGGTGCCTTGC
h_SOD2_for	GCACTAGCAGCATGTTGAGC
h_SOD2_rev	TTGATGTGAGGTTCCAGGGC
h_UCP1_for	CGCAGGGAAAGAAACAGCAC
h_UCP1_rev	TTCACGACCTCTGTGGGTTG
h_VEGF_for	ACAACAAATGTGAATGCAGACCA
h_VEGF_rev	GAGGCTCCAGGGCATTAGAC
